# *In silico* studies of M^pro^ and PL^pro^ from SARS-CoV-2 and a new class of cephalosporin drugs containing 1,2,4-thiadiazole

**DOI:** 10.1007/s11224-022-02036-5

**Published:** 2022-09-10

**Authors:** Cássia Pereira Delgado, João Batista Teixeira Rocha, Laura Orian, Marco Bortoli, Pablo Andrei Nogara

**Affiliations:** 1 Departamento de Bioquímica e Biologia Molecular, Universidade Federal de Santa Maria (UFSM), Santa Maria, RS 97105-900 Brazil; 2 Dipartimento di Scuenze Chimiche, Università degli Studi di Padova, Via Marzolo 1, 35131 Padua, Italy; 3 Institut de Química Computacionali Catàlisi (IQCC), Departament de Química, Facultat de Ciències, Universitat de Girona, C/M. A. Capmany 69, 17003 Girona, Spain

**Keywords:** Cephalosporins, Drug repurposing, Computational docking, 1,2,4 thiadiazoles, DFT calculations, Molecular dynamics

## Abstract

The SARS-CoV-2 proteases M^pro^ and PL^pro^ are important targets for the development of antivirals against COVID-19. The functional group 1,2,4-thiadiazole has been indicated to inhibit cysteinyl proteases, such as papain and cathepsins. Of note, the 1,2,4-thiadiazole moiety is found in a new class of cephalosporin FDA-approved antibiotics: ceftaroline fosamil, ceftobiprole, and ceftobiprole medocaril. Here we investigated the interaction of these new antibiotics and their main metabolites with the SARS-CoV-2 proteases by molecular docking, molecular dynamics (MD), and density functional theory (DFT) calculations. Our results indicated the PL^pro^ enzyme as a better *in silico* target for the new antibacterial cephalosporins. The results with ceftaroline fosamil and the dephosphorylate metabolite compounds should be tested as potential inhibitor of PL^pro^, M^pro^, and SARS-CoV-2 replication *in vitro*. In addition, the data here reported can help in the design of new potential drugs against COVID-19 by exploiting the S atom reactivity in the 1,2,4-thiadiazole moiety.

**Supplementary Information:**

The online version contains supplementary material available at 10.1007/s11224-022-02036-5.

## Introduction

The global respiratory pandemic COVID-19 (coronavirus disease 2019) is caused by the severe acute respiratory syndrome coronavirus 2 (SARS-CoV-2), which remains afflicting millions of people. Although the majority of infected people are asymptomatic or present mild symptoms [1–3], the severe cases can evolve to pneumonia, heart injury, kidney failure, and central nervous system symptoms (encephalitis, seizures) and death [1, 4–6].

Cysteine proteases are one of the four main groups of peptide-bond hydrolases. They all use a S^−^ anion (thiolate) of a cysteine (Cys) side chain as the nucleophile in the hydrolysis of the peptide bond [7]. The cysteinyl proteases are found in all forms of life and can also be codified by single-stranded RNA viruses. In vertebrates, cysteinyl proteases can mediate a wide variety of physiological and pathological processes. In different viruses, cysteinyl proteases display important roles in the virion formation, release, and entry into the host cells. Typically the cysteinyl proteases metabolize the formation of critical viral proteins inside the host cells [8–10].

Coronaviruses (CoVs) are a large group of enveloped, single-stranded, positive-sense RNA viruses that encode large replicase polyproteins that are processed by viral peptidases to generate proteins involved in viral replication [7, 10]. The SARS-CoV-2 papain-like protease (PL^pro^ or non-structural protein 5, nps5) and 3C chymotrypsin-cysteine-like peptidase or main protease (M^pro^ or nps3) process post-translationally the viral pp1a and pp1ab polyproteins in non-structural proteins. The cysteinyl residues found in the catalytic moieties of the M^pro^ and PL^pro^ are directly involved in the hydrolysis of specific peptide bonds presented in the large pp1a and pp1ab polyproteins [11, 12]. Consequently, the cysteine-proteases from coronavirus (MERS-CoV, SARS-CoV, and SARS-CoV-2) have been considered targets for the repositioning of therapeutically approved drugs or for the development of new agents [13–17].

Since the beginning of the pandemic, extensive efforts have been done in the search for SARS-CoV-2 proteases’ inhibitors [15]. For instance, the organochalcogen drugs ebselen, disulfiram, and tideglusib have been demonstrated to inhibit the M^pro^ from SARS-CoV-2 *in vitro* [15]. The inhibitory capacities of some of these molecules have also been analyzed and confirmed *in silico* [18, 19]. From the ~ 10,000 molecules investigated by Jin *et al*. [15], tideglusib was the only one containing the 1,2,4 thiadiazole group, and it inhibited the M^pro^ with a good potency [15]. However, the authors did not comment about the *in silico* or *in vitro* inhibition of Sars-Cov-2 M^pro^ by other molecules containing the 1,2,4-thiadiazole moiety.

**Fig. 1:**
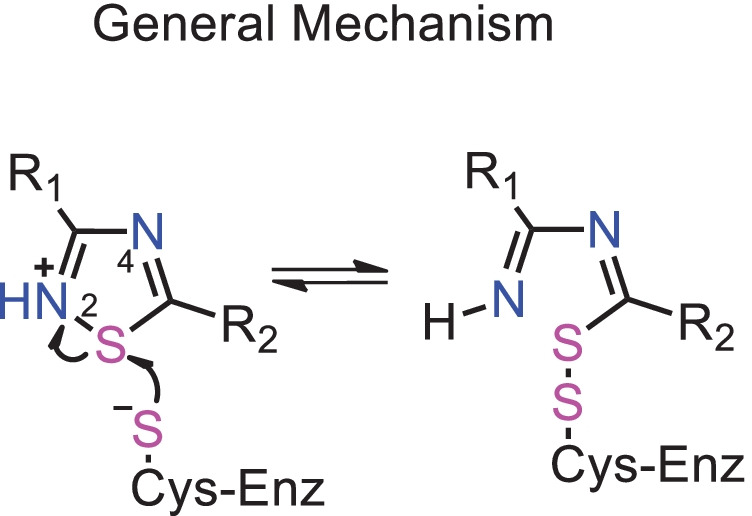
General structure and proposed inhibitory mechanism of Cys enzymes (Enz) by1,2,4-thiadiazoles molecules. R_1_ and R_2_ are organic substituents

The inhibitory mechanism of tideglusib against M^pro^ has not been investigated in detail. But the 1,2,4 thiadiazole moiety has been reported to inhibit cysteinyl proteases, for instance, papain and cathepsins B, L, and K [20–22]. The S atom of 1,2,4 thiadiazole behaves as an electrophilic center, while the thiol (–SH) of the Cys proteases attacks the sulfur atom of the ring to form a disulfide bond with concomitant ring opening (**Fig.** **1**) [21–23]. Recently, Sarkar *et al*. demonstrated that the compound RRA2 exhibited mycobactericidal activity against the intracellular satage *Mycobacterium bovis* and *Mycobacterium tuberculosis *at the* micromolar range* [24].

Recently, Kumar *et al*. [25] have performed an *in silico* search for potential repurposing candidate drugs in the Korea Chemical Bank drug reuse database (KCB-DR). They have indicated some putative inhibitors of M^pro^, including ceftaroline fosamil (a drug containing 1,2,4-thiadiazole functional group) [25]. The virtual screening, molecular dynamics (MD) simulations, and binding-free energy approaches demonstrated the interaction between ceftarolinefosamil, forming hydrogen bonds with active site residues in M^pro^, such as His41. However, the authors did not investigate the possible mechanism of inhibition nor the interaction of the S atom from ceftaroline fosamil with the cysteinyl residue (Cys 145) in the enzyme’s active site.

Since the 1,2,4-thiadiazole-containing molecule tideglusib has been report to inhibit the SARS-CoV-2 M^pro^ *in vitro* [15], here we did a systematic search for compounds containing the moiety 1,2,4-thiadiazole. In the Drug Bank database, from searches for structural similarity of compounds containing the functional group, 1,2,4-thiadiazole, to optimize the potential discovery of new antiviral drugs from previously approved therapeutic agents by the FDA (Food and Drug Administration) that could be further studied for the potential repositioning in the treatment of COVID-19.

**Fig. 2:**
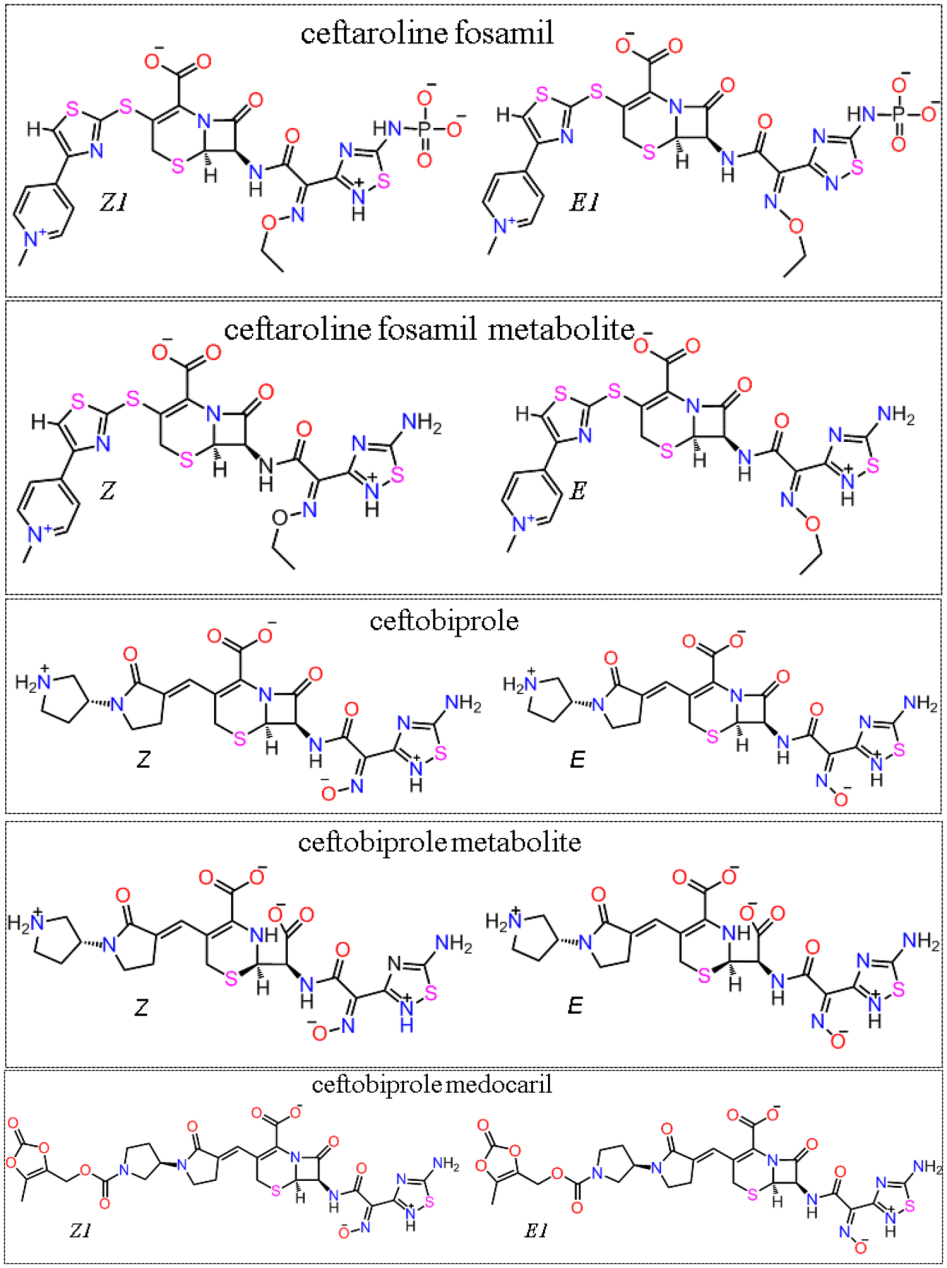
Chemical structure and isomers of 1,2,4-thiadiazoles drugs and their active metabolites. The approved ceftarolinefosamil and ceftobiprole, and an experimental drug, ceftobiprole medocaril. For ceftarolinefosamil and ceftobiprole medocaril, isomers *Z*1 and *E*1 are the most abundant protonation forms, at physiological pH between 7.0 and 7.4, (determined in the Marvin Sketch program). The other forms, and proportions at physiological pH range, are presented in the Supporting Information

Our search for drugs containing the 1,2,4-thiadiazole functional group retrieved two approved drugs (ceftaroline fosamil and ceftobiprole) and one in the experimental phase (ceftobiprole medocaril) **Fig.** **2**. They are a new generation of broad-spectrum cephalosporins in late stages of development with activity against methicillin-resistant *Staphylococcus aureus* (MRSA) [26, 27]. The presence of the 1,2,4-thiadiazole moiety has been reported to facilitate the antibiotic permeation inside Gram-negative bacteria and the transpeptidase activity [27, 28]. In addition, these drugs are already in clinical use for the treatment of human respiratory tract infections and pneumonias [26]. Of therapeutic significance, pharmacokinetic studies have indicated that intravenous administration of ceftaroline fosamil, ceftobiprole, and ceftobiprole medocaril resulted in micromolar plasma and epithelial lining fluid (ELF) concentrations of their active metabolites [29–32]. One of the advantages of repurposing drugs already approved for clinical use is the availability of data about their toxicity and their concentration found in relevant body fluids [33, 34].

Thus, in this work we perform *in silico* molecular docking analyses, density functional theory (DFT) calculations, and molecular dynamics (MD) to propose new M^pro^ and PL^pro^ inhibitors, as well as to explain the mechanisms of enzyme inhibition at the molecular and atomic levels. The latter analyses are fundamental to assess the role of the distinct molecular moieties for improved and rational drug design.

## Materials and methods

### 1,2,4-thiadiazole containing drugs and metabolites

The Drug Bank (www.go.drugbank.com) [35] was utilized to search for FDA-approved drugs containing 1,2,4-thiadiazole functional group [35]. The ceftaroline fosamil, ceftobiprole, and their respective metabolites were retrieved as approved drugs, while the ceftobiprole medocaril is still in the experimental list. They were all considered in our *in silico* studies (including their *Z* and *E* isomers) to verify if they might be M^pro^ and PL^pro^ potential inhibitors.

### Docking simulations

Auto Dock Vina was used for the docking simulations [36], with exhaustiveness of 50, according to a previous study [19]. The M^pro^ and PL^pro^ crystallographic structures were obtained from the Protein Data Bank (PDB) with the codes 6LU7 and 7JN2, respectively. Water, ions, ligands, and other molecules were removed from the protein structures; then, the hydrogen atoms were added using the CHIMERA program, followed by 100 steps of energy minimization [37]. The M^pro^ grid box of size 25 × 35 × 25 Å was centered on the active site from chain A (− 14.04, 17.44, 66.22). For the PL^pro^, the docking grid box was centered on the active site (39.64, 30.68, 1.66; size: 20 × 20 × 20 Å) and in the Zn binding site (82.40 × 26.32 × − 0.62; size: 20 × 20 × 20 Å), both from chain A. The three-dimensional model of ceftaroline fosamil, ceftobiprole, ceftobiprole medocaril, and their metabolites was created with Avogadro and MOPAC (PM6 method) [38–40], using the dielectric constant of water (78.4), and taking into account the physiological pH (7.0 to 7.4) as determined by in the Marvin Skecth 17.21.0, ChemAxon program (see Supporting Information (SI) **Figs.** **S14****–****S16**) (ChemAxon—Software Solutions and Services for Chemistry & Biology [41]). In addition, both *Z* and *E* isomers of the drugs ceftaroline fosamil and ceftobiprole medocaril were considered in this study. These details are very important and can affect the predicted binding pose in molecular docking simulations. For each molecule, the 20 best conformers (in terms of ∆G) were analyzed in the Discovery Studio Visualizer program [19, 42]. Two conformers were chosen, i.e., the conformer with the largest negative binding energy [43] and the conformer with the shorter S···S interaction. The conformers of 1,2,4-thiadiazole-containing drugs and metabolites which displayed the best interaction with the Cys residues from M^pro^ and PL^pro^ (in terms of S···S distances and ∆*G*) were highlighted. Specifically, the distance between the S atom (ligand) to the S atom (Cys) was considered an indicator of potential covalent bond formation between 1,2,4-thiadiazole compounds and metabolites with the enzymes.

In total, 14 molecules were tested, derived from the drugs ceftarolinefosamil, ceftobiprole, and ceftobiprole medocaril, including their isomers and protonated forms in their predominant pH states, SI **Figs.** **S14****–****S16**. Among them, 10 will be discussed in this article; the others are in the supplementary material section. Isomers (*Z*,*E*)1 indicate the most populous protonation state, and the (*Z*,*E*)2, the least populous.

### Density functional theory (DFT) calculations

All density functional theory (DFT) calculations were carried out using the Amsterdam Density Functional (ADF) program [44, 45]. Scalar relativistic effects were taken into account using the zeroth-order regular approximation (ZORA) [46]. The OLYP density functional was used, in combination with the TZ2P basis set, according to the literature [47]. The softness (*σ* = 1/*η*,*η* = [*E*(LUMO) − *E*(HOMO)]/2) was computed according to LoPachin *et al*. [48], using the Hirshfeld charges [49], which have a good overall reactivity prediction performance [50, 51].

### Molecular dynamics

Molecular dynamics simulations were run for the two main compounds, namely, ceftaroline fosamil and ceftaroline fosamil dephosphorylated metabolite, employing AMBER 2021 [52]. The complexes were treated using the AMBER ff14SB force field for the protein residues PL^pro^ and the generalized AMBER force field (GAFF) to define the ligands’ parameters. To simulate the Zn^2+^ atom present in PL^pro^, the four cysteine residues directly bonded to zinc and the zinc atom itself were modeled employing the Zinc AMBER Force Field (ZAFF) [53]. The structures were solvated in an octahedral box of TIP3P water molecules. Starting from the best pose obtained via the docking procedure and after energy minimization, heating to 310 K at constant volume and temperature was performed over 60 ps using the Langevin thermostat.

Afterward, equilibration at constant temperature (310 K) and pressure (1 bar, Berendsenbarostat) was conducted for 60 ps using weak restraints (2 kcal mol^−1^ Å^−2^) on the protein–ligand complex and then for 2 ns without any restraint, followed by 200-ns production runs from the MD trajectories, 1000 frames taken at 0.2-ns intervals were extracted and employed in the calculation of RMSDs and RMSFs.

## Results and discussion

The docking of ceftobiprole and all the *Z* and *E* isomers of ceftaroline fosamil, ceftaroline fosamil dephosphorylated metabolite, ceftobiprole, ceftobiprole medocaril, and ceftobiprole metabolites in their conformations with the shortest S···S distance are presented in the main text, **Fig.** **4** and **Table** **1**. The data of the best energy conformers were included in the supplementary information (**SI**). The selection of conformers was based on the predominant conformations found between the pH values of 7.0 to 7.4 (the percentages of occurrence of each conformer is presented in **Table** **S2**). Ceftaroline fosamil isomers (*Z*,*E*)2 and ceftobiprole medocaril isomers (*Z*,*E*)2 are depicted in the SI. It is emphasized that the *Z* and *E* isomers centered in the oxime group can interfere in the way drugs and metabolites interact with the active sites of the proteases under study. The configuration of the *E* isomer is sterically closer to the functional group under study, 1,2,4-thiadiazole, **Fig.** **2**.

**Fig. 3:**
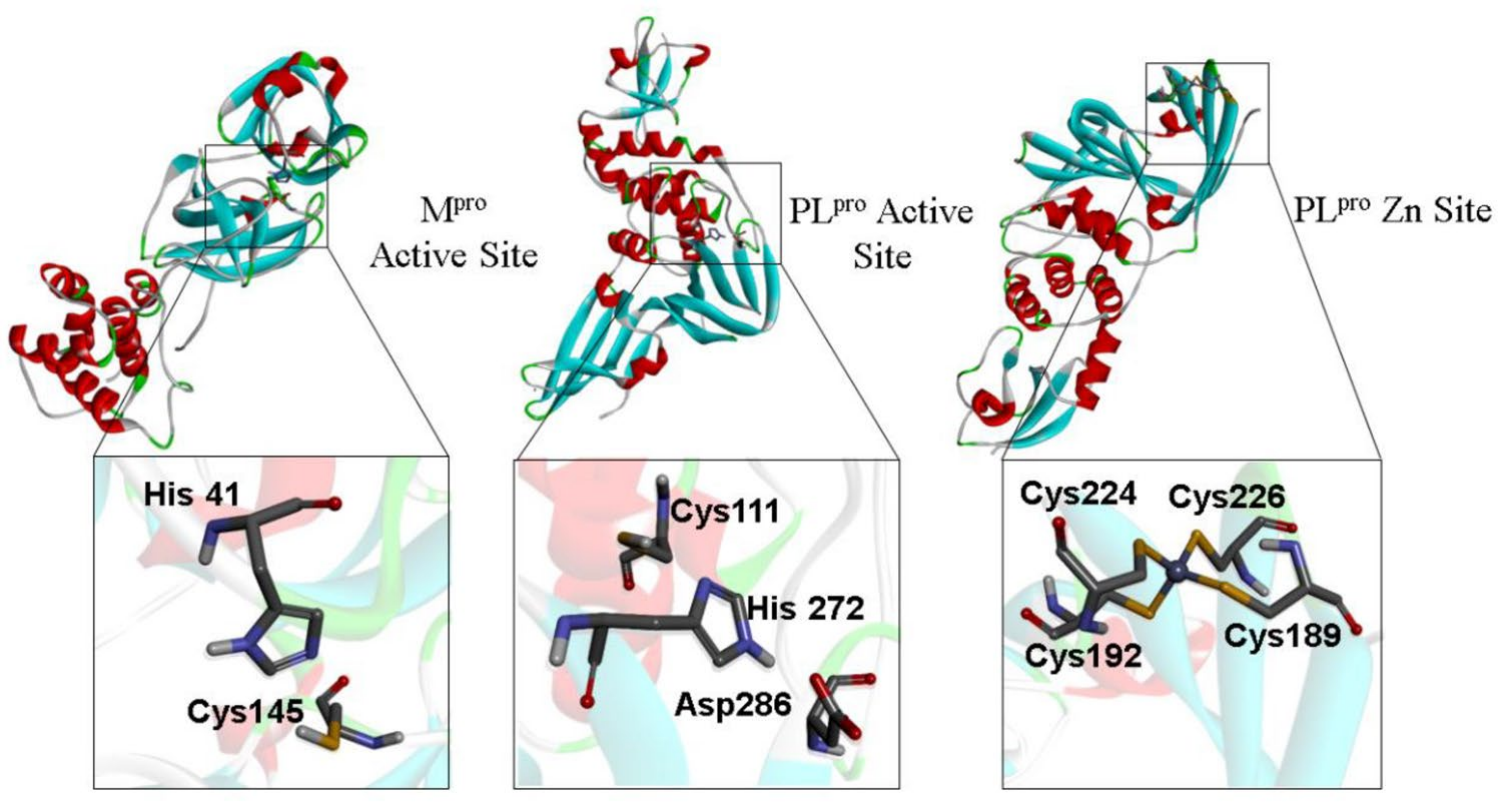
Target cysteinyl residues in the SARS-CoV-2 M^pro^ active site (Cys145), PL^pro^ active site (Cys11), and Zn binding site (Cys189, Cys192, Cys224, and Cys226) are depicted in the figure. **A** M^pro^ active site (6lu7), **B** PL^pro^ active site, and **C** PL.^pro^ Zn site (7jn2)

Taking into consideration the proposed inhibitory mechanism of 1,2,4-containing molecules against cysteinyl proteases (**Fig.** **1**), which involves the nucleophilic attack of the thiolate of cysteinyl residues on the S atom of the 1,2,4-thiadiazole heterocycle (**Fig. ****2**), here we have emphasized the *in silico* interaction of cysteinyl residues from the active site of M^pro^ and PL^pro^, and the 4 cysteinyl residues coordinating PL^pro^ Zn (the Zn binding site) with the S atom of the 1,2,4-thiadiazole moiety in the new class of cephalosphorin antibiotics. Specifically, the distances between Cys S···S (1,2,4-thiadiazole) were evaluated to understand the potential role of the cephalosporin antibiotics as inhibitors of SARS-CoV-2 proteases M^pro^ and PL^pro^, **Fig.** **3**.

### M^pro^ Cys145 interaction with 1,2,4-thiadiazole containing drugs and metabolites

**Fig. 4:**
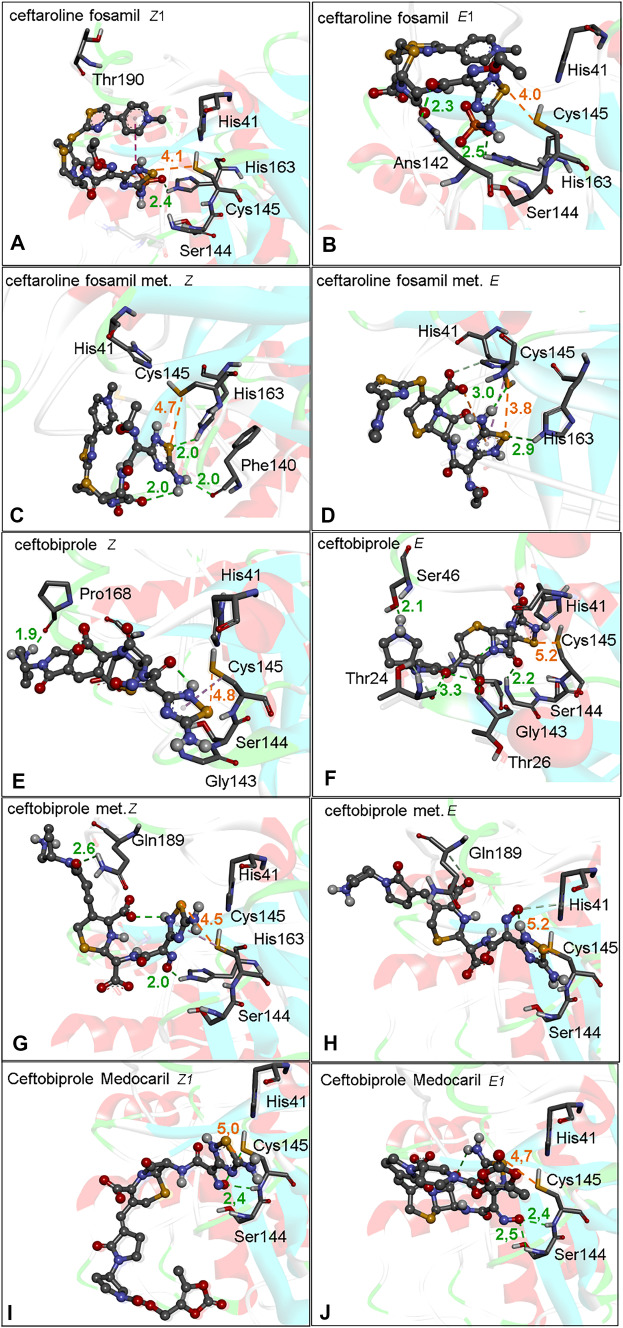
M^pro^ docking with 1,2,4-thiadiazole containing drugs and their metabolites. **A** Ceftaroline fosamil isomer *Z1*. **B** Ceftaroline fosamil isomer *E1*. **C** Ceftaroline fosamil dephosphorylated metabolite isomer *Z*. **D** Ceftaroline fosamil dephosphorylated metabolite isomer *E*. **E** Ceftobiprole isomer *Z*. **F** Ceftobiprole isomer E. **G** Ceftobiprole metabolite isomer *Z*. **H** Ceftobiprole metabolite isomer *E*. **I** Ceftobiprole medocaril isomer *Z1*. **J** Ceftobiprole medocaril isomer *E1*. Distances are shown in Å

M^pro^ is the main integrant of the proteolytic processing machinery of SARS-CoV-2 and is greatly conserved in coronaviruses [54]. The M^pro^ cleaves the polyproteins (pp) 1a and 1ab from SARS-CoV-2 into 16 distinct proteins, which are essential for the formation of viral replication complexes [55]. Similar to other cysteine proteases, the active site of M^pro^ contains a Cys-His catalytic dyad that has a critical role in the enzyme structure and coordinates the hydrolyzes of the peptide bonds at specific sites of the polyproteins 1a and 1ab chains [56]. The His41 accepts the proton from the thiol group of Cys145; consequently, the nucleophilicity of Cys145 residue increases considerably [57]. The covalent blockage of the thiol/thiolate moiety of Cys145 inhibits the M^pro^ proteolytic function. The binding poses obtained from the docking analyses on the M^pro^ active site demonstrated that the sulfur atom from all 1,2,4-thiadiazoles-containing drugs and metabolites interacted with the thiol/thiolate group of Cys145 and with surrounding residues (**Fig.** **4****A–J**).

The ceftaroline fosamil isomers (*Z*,*E*)1 showed similar bond position and S···S interactions (**Fig.** **4****A, B**), while the binding poses of ceftaroline fosamil dephosphorylated metabolites *E* and *Z* and S···S distances were different (3.8 Å and 4.7 Å, respectively (**Fig.** **4****C, D**). Thus the removal of the phosphate group influenced the binding poses of Z and E dephosporylated ceftaroline fosamil isomers in the active site of M^pro^.

For the ceftobiprole isomers, comparable binding poses were predicted, but with a little different distances from the Cys145. In fact, the (Cys)S···S(thiadiazole) interaction was shorter for the *Z* isomer (4.8 Å) than for the *E* (5.2 Å) (**Fig.** **4****E, F**). For the ceftobiprole metabolite isomers, a similar binding pose of thiadiazole was observed. However, ceftobiprole metabolite *Z* (4.5 Å) presented a shorter S···S interaction than the isomer *E* (5.2 Å) (**Fig.** **4****G, H**).

For the ceftobiprole medocaril isomers, slightly different S···S distances were observed; the *Z1* (5.0 Å) presented a little longer interaction than did the isomer *E1* (4.6 Å) (**Fig.** **4**I, **J**). The analysis of the Cys145S···S1,2,4-thiadiazole compounds interaction that the *E1* isomer from the dephorylated ceftaroline fosamil presented the shortest distance (**Table** **1**).

In addition, it is important to note that H-bonds between Ser144, His163, Gln189, Asn142, and Cys145 residues with the ligands participated in the stabilization of M^pro^-ligand complexes. Hydrophobic interactions between the 1,2,4-thiadiazole can also have significant contribution for the stabilization of M^pro^-ligand complexes, as a recently demonstrated by Kumar *et al*. [25]. They reported the *in silico* interactions between ceftaroline fosamil and the amino acid residues in or near to the active site of M^pro^. In addition to the hydrogen bonds between Thr24, Thr25, His41, and Thr45 with the antibiotic, they indicated hydrophobic interactions between Cys44, Ser46, Met49, Met165, Arg188, Gln189, Thr190, Ala191, Gln192 with ceftaroline fosamil. In short, the secondary interactions between amino acid residues near the active site with the cephalosporine derivatives have also important contributions to the overall stability of the protease-compound complex.

Among the conformers with the largest negative binding energy (**Table** **2**), only the dephosphorylated ceftaroline fosamil *Z* isomer, ceftobiprole *E*, ceftobiprole metabolite *Z*, and ceftobiprole medocaril isomer *E*1 showed S···S interaction; however, their S···S distances were longer than those observed with the conformers presented in **Table** **1**. A similar H-bond pattern was observed for these conformers, with distances varying from 1.8 to 3.0 Å with Thr190, Ser144, His163, Thr26, Asn142, Glu166, and Cys145.

**Table 1:**
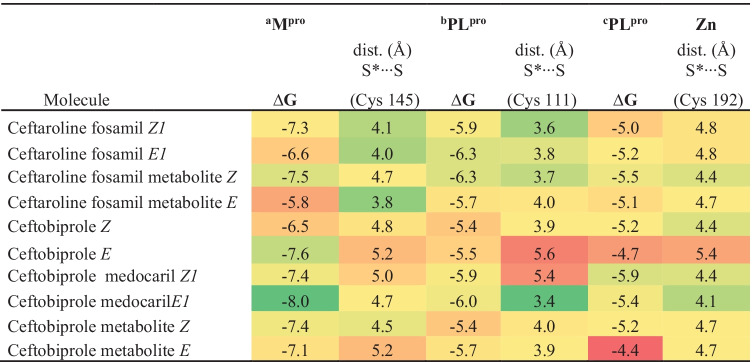
Predicted binding free energies (∆*G*, kcal·mol^−1^) between M^pro^ and PL^pro^ and PL^pro^ Zn binding site, with 1,2,4-thiadiazole containing drugs with the select conformer presenting the most favorable S···S interaction distances

S*···S indicates the distance interaction in (Å) of the electrophile center of the ligand (i.e., the sulfur atom of the 1,2,4 thiadiazole heterocycle with the sulfur atom of the cysteinyl residues of M^Pro^. Distance (in Å) of the thiol from ^a^Cys145, ^b^Cys111, and ^c^Cys192 to the ligand of the S from the 1,2,4-thiadiazole heterocycle. The green, yellow, and red colors indicate a favorable, intermediate, and less favorable interaction, respectively

**Table 2:**
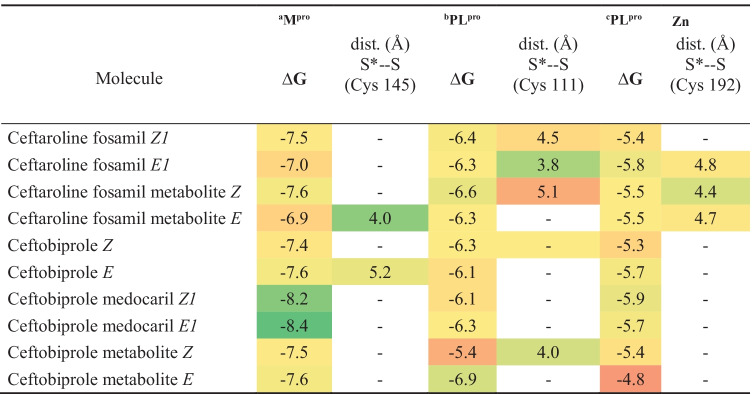
Predicted binding free energies (∆*G*, kcal·mol^−1^) between M^pro^ and PL^pro^ and PL^pro^ Zn binding site, with 1,2,4-thiadiazole containing drugs with the largest negative ∆*G* binding energy

S*···S indicates the distance interaction (in Å) of the electrophile center of the ligand (i.e., the sulfur atom of the 1,2,4 thiadiazole heterocycle with the sulfur atom of the cysteinyl residues of M^pro^. Distance (in Å) of the thiol from ^a^Cys145, ^b^Cys111, and ^c^Cys192 to the ligand of the S from the 1,2,4-thiadiazole heterocycle. The green, yellow, and red colors indicate a favorable, intermediate, and less favorable interaction, respectively

### 1,2,4-thiadiazole containing drugs and metabolites interaction with PL^pro^

In the PL^pro^, the Cys111 from the catalytic triad contains the nucleophilic center that directly participates in the cleavage of the peptide bond of polyproteins from SARS-CoV-2. The His272 and Asp286 residues participate in the catalysis as acid–base pairs that promote the thiol deprotonation of Cys111. The resulting thiolate has enhanced nucleophilicity [11, 58]. The blockage of the thiol moiety of Cys111 is an important strategy to inhibit PL^pro^.

**Fig. 5:**
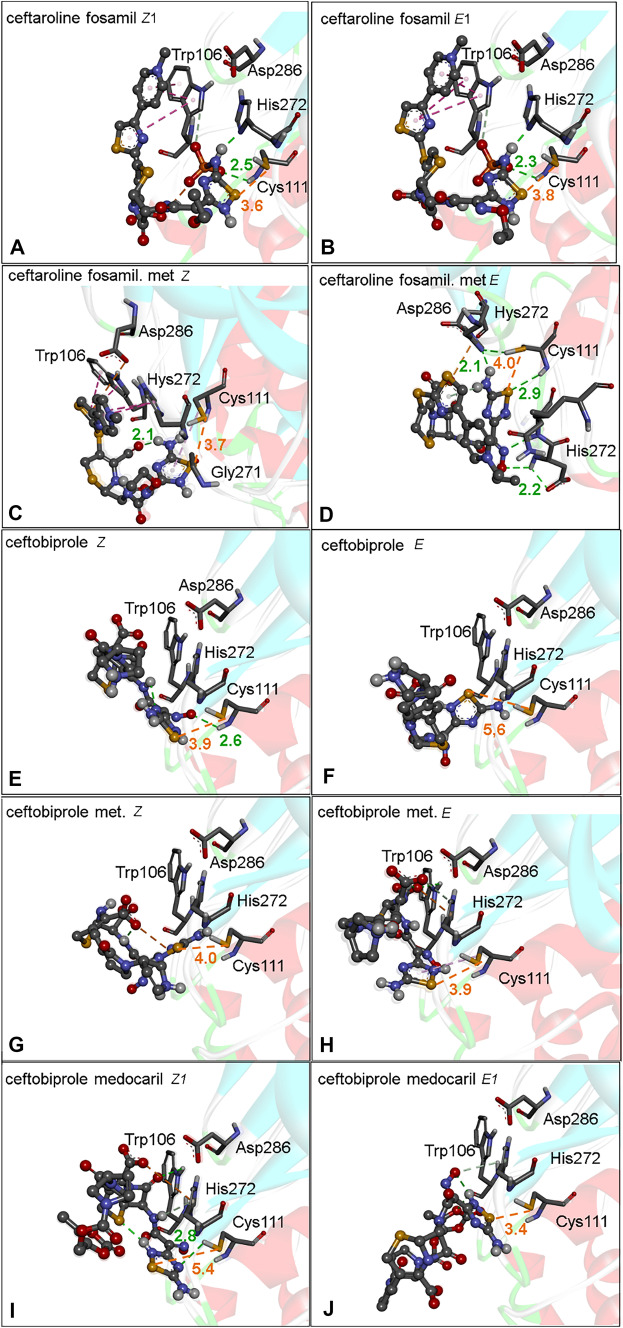
PL^pro^ docking with 1,2,4-thiadiazole containing drugs and their metabolites. **A** Ceftaroline fosamil isomer *Z1*. **B** Ceftaroline fosamil isomer *E1*. **C** Ceftaroline fosamil dephosphorylated metabolite isomer *Z*. **D** Ceftaroline fosamil dephosphorylated metabolite isomer *E*. **E** Ceftobiprole isomer *Z*. **F** Ceftobiprole isomer E. **G** Ceftobiprole metabolite isomer *Z*. **H** Ceftobiprole metabolite isomer *E*. **I** Ceftobiprole medocaril isomer *Z1*. **J** Ceftobiprolemedocaril isomer *E1*. Distances are shown in Å

The binding poses obtained from the docking studies focusing on the PL^pro^ active site demonstrated that the sulfur atom of 1,2,4-thiadiazole-containing drugs and metabolites can interact with the thiol group of Cys111 residue (**Fig.** **5****A–J**).

The ceftaroline fosamil isomers (*Z*,*E*)1 displayed similar bonding poses and a little difference in the distance of S···S interactions (*Z*1 ~ 3.6 Å and *E*1 ~ 3.8 Å). The distances of the S···S interactions in the dephosphorylated ceftaroline fosamil metabolite were more favorable in the *Z* (3.7 Å) than in the *E* isomer (4.0 Å) (**Fig.** **5**A–**D**).

The ceftobiprole isomers interacted with different binding poses and different S···S interaction distances with the active site of PL^pro^, where the *Z* isomer (3.9 Å) has a shorter distance than the *E* (5.6 Å) isomer (**Fig.** **5****E, F**). In the ceftobiprole metabolite, the shortest interaction was observed for the isomer *E* (3.9 Å) (**Fig.** **5****G, H**). For the ceftobiprole medocaril, a shorter interaction was observed in the isomer *E*1 (3.4 Å) than in isomer *Z*1 (5.4 Å) (**Fig.** **5****I, J**).

In view of the proposed mechanism for the inhibition of the proteases depicted in **Fig.** **1**, it is important to note that the H-bonds between His272 and Cys111 are critical for stabilizing the PL^pro^-ligand complexes. Indeed, the docking data here presented confirm the importance of the interaction between His 272 and Cys111 (**Fig.** **5**). Similar patterns of H bond interactions were observed for the energetically most stable conformers, with distances ranging from 1.8 to 3.3 Å between amino acids surrounding the active site (for instance, Ans109, His272, and Cys111). The binding poses for these interactions are shown in SI **Figs.** **S7** and **S8**.

### 1,2,4-thiadiazole containing drug and metabolite interaction with PL^pro^ Zn binding site

While the active sites of M^pro^ and PL^pro^ have only one Cys as a potential target for electrophilic inhibitors, the Zn binding site in the PL^pro^ is composed of a Zn ion coordinated with four Cys residues (Cys189, Cys192, Cys224, and Cys226). This type of Cys-rich motif structure is found in many metalloproteins, for instance, in zinc finger–containing proteins [59, 60]. Theoretically, the inhibition of the PL^pro^ by the oxidation of the cysteinyl residues located at the Zn binding site by 1,2,4 thiadiazole containing molecules has not been elucidated yet.

**Fig. 6:**
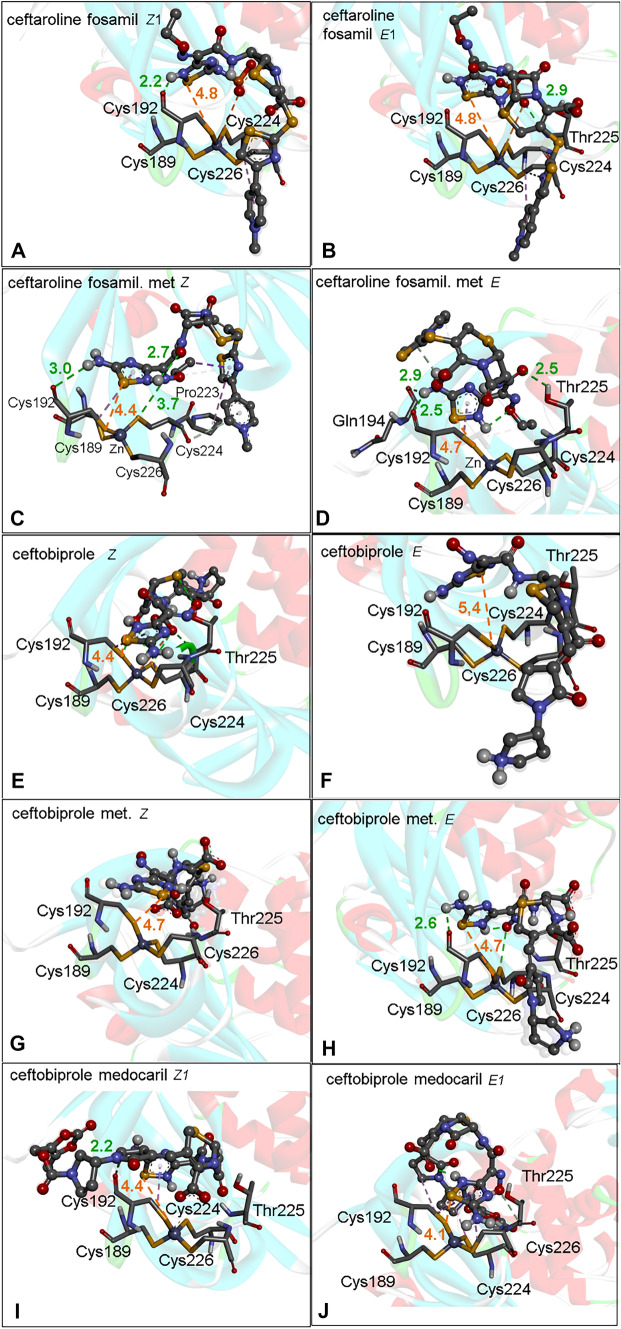
PL^pro^ Zn site docking with 1,2,4-thiadiazole containing drugs and their metabolites. **A** Ceftaroline fosamil isomer *Z1*. **B** Ceftaroline fosamil isomer *E1*. **C** Ceftaroline fosamil dephosphorylated metabolite isomer *Z*. **D** Ceftaroline fosamil dephosphorylated metabolite isomer *E*. **E** Ceftobiprole isomer *Z*. **F** Ceftobiprole isomer E. **G** Ceftobiprole metabolite isomer *Z*. **H** Ceftobiprole metabolite isomer *E*. **I** Ceftobiprole medocaril isomer *Z1*. **J** Ceftobiprole medocaril isomer *E1*. Distances are shown in Å

The PL^pro^ inhibition can occur via the ejection of zinc ion from the PL^pro^ Zn binding site of SARS-CoV-2. Accordingly, the recent study of Sargsyan *et al*. has indicated that ebselen and disulfiram bind covalently to Cys residues of the PL^pro^ Zn binding site [61]. In the present study, the binding poses obtained from the docking simulations focusing on the Zn site demonstrated that the sulfur atom of the 1,2,4-thiadiazole-containing drugs and metabolites interacted preferentially with the thiol group of Cys192 (**Fig.** **6****A–J**).

The ceftaroline fosamil isomers (*Z*,*E*)1 exhibited similar binding poses and distances between the sulfur atoms from the 1,2,4 thiadiazole moiety and the Cys192 (4.8 Å), while the dephosphorylated metabolite isomer binding pose and S···S distances were a little different, ranging from 4.7 (*E*) to 4.4 Å (*Z*) (**Fig.** **6****A–D**).

The ceftobiprole isomers exhibited distinct binding poses and S···S distances. The interaction with Cys192 was shorter for the *Z*1 (4.3 Å) than for the *E*1 isomer (5.3 Å) (**Fig.** **6****E, F**). In the case of ceftobiprole metabolites, identical distances were obtained with the *Z* and *E* isomers (4.7 Å) (**Fig.** **6****G, H**). Regarding the ceftobiprole medocaril isomers, the sterical binding poses was different and the S···S interaction distances in the *Z* (4.4 Å) and *E* isomer (4.1 Å) were slightly different (**Fig.** **6****I–J**).

It is important to note that the H-bonds with Thr225 and Cys192 help to stabilize the PL^pro^ Zn-ligand complexes. Consequently, the interactions described here for the antibiotics and metabolites containing the 1,2,4-dithiazolee moiety may be involved in the potential mechanism of inhibition of PL^pro^ via the ejection of the Zn ion from the Zn binding site. Accordingly, the nucleophilic attack of the thiolate from Cys192 at electrophile sites found in “zinc ejectors” molecules can explain their inhibitory effects in the SARS-CoV-2 PL^pro^ as previously demonstrated by Sargsyan *et al*. [61]. Considering the conformers with the largest negative Δ*G*, ceftaroline fosamil *E*1, dephosphorylated ceftaroline fosamil metabolite *Z*, ceftobiprole *Z*, and ceftobiprole medocaril (*Z*,*E*)1 demonstrate optimal S···S interactions to form a covalent bond (**Table** **2** and **Figs.** **S9** and **S10**).

The best conformers with better interactions (in terms of Δ*G*, value) with the PL^pro^ Zn binding site exhibited (Cys192) S···S (1,2,4thiadiazole) distances ranging between 4.8 and 5.1 Å (**Table** **2**).

### Reaction between a thiolate and 1,2,4-thiadiazoles

According to the docking studies, the 1,2,4-thiadiazole ring might be the reactive center of the antibiotic drugs. Thus, we hypothesized that this moiety might react with the catalytic or structural Cys residues forming a stable adduct via a disulfide bond (S–S). We studied the reactivity and computed the reaction energies modeling the process with 1,2,4-thiadiazole ring (tdz) and a methylthiolate (MeS^−^) (low molecular weight thiols are often used as a simple model of Cys residues or other biological thiols like GSH in DFT calculations [62–64]. The 3,5-dimethyl-1,2,4-thiadiazole (tdzMe_2_) and 3-methyl-1,2,4-thiadiazol-5-amine (tdzMeN) were used as models of the antibiotic drugs. Furthermore, the reactivities of the corresponding protonated molecules ([tdzHMe_2_]^+^and [tdzHMeN]^+^) were also investigated.

To better understand the reactivity, Hirshfeld charges were computed and the softness analysis was done (**Table** **3**). The results suggest that the reactions of MeS^−^ with protonated thiadiazoles ([tdzHMe]^+^ and [tdzHMeN]^+^) are more favorable than those with the neutral forms. Also according to the hard and soft, acids and bases (HSAB) theory, soft bases (MeS^−^) prefer to react with soft acids ([tdzHMe]^+^ and [tdzHMeN]^+^) [48]. The S partial charge analysis from the [tdzHMe_2_]^+^ indicated that this atom is more electrophilic in the protonated than in neutral form (**Table** **3**).

**Table 3:** Hirshfeld charges, frontier orbital energies (eV), and softness (*σ*) of the reactants

Phase	Reactants	S charge	HOMO	LUMO	Softness
Gas	MeS^−^	− 0.737	1.741	4.062	0.861
	tdzMe_2_	0.150	− 6.293	− 1.804	0.445
	[tdzHMe_2_]^+^	0.322	− 11.976	− 7.652	0.462
	tdzMeN	0.114	− 5.534	− 1.298	0.472
	[tdzHMeN]^+^	0.271	− 10.922	− 6.923	0.500
Water	MeS^−^	− 0.847	− 4.776	− 0.375	0.454
	tdzMe_2_	0.184	− 6.458	− 1.965	0.445
	[tdzHMe_2_]^+^	0.381	− 7.495	− 3.090	0.454
	tdzMeN	0.130	− 5.662	− 1.421	0.471
	[tdzHMeN]^+^	0.307	− 6.461	− 2.462	0.500

Softness (eV^−1^): *σ* = 1/*η*; hardness (eV): *η* = [E(LUMO) − E(HOMO)]/2. Level of theory: (COSMO)-ZORA-OLYP/TZ2P

As shown in **Table** **4**, the reaction between the protonated [tdzHMe_2_]^+^ and MeS^−^ produced an adduct with a disulfide bond and in an open ring conformation (entries A and B). In the gas phase, the reaction energy was more negative than in water because the charged reactants are much less stabilized in the gas phase than in water [65, 66].We studied this reaction also using the cysteinate as a nucleophile (C and D), and the energy values in gas and condensed phase also presented this large difference.

**Table 4:**
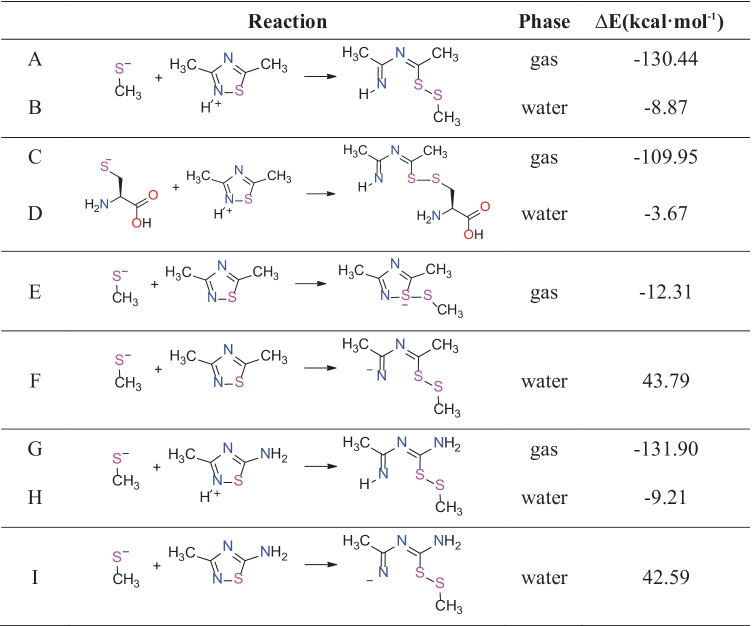
Reaction energies. Level of theory, ZORA-OLYP/TZ2P

In the gas phase, using the neutral thiadiazole (tdzMe_2_) as substrate, we obtained a three-center intermediate (TCI) [tdzMe-SMe]^−^ instead of the open ring product [tdzMe_2_-SMe]^−^ which was recovered in water (E and F). The TCI-[tdzMe_2_-SMe]^−^ presented negative reaction energy while the [tdzMe_2_-SMe]^−^ formation in water shows positive reaction energies, suggesting an endergonic process.

Finally, we used the [tdzHMeN]^+^ model, in which the methyl group at position 5 of the 1,2,4-thiadiazole was replaced by an amine group (in similar way to that found in the antibiotic drugs), to compute the reaction energies (G and H). As previously observed, there was a significant difference between the energies in gas and condensed phase. Anyway, they are both energetically favorable. In contrast, the reaction with the neutral tdzMeN molecule was found to be an endergonic process (I), suggesting that the protonation of the 1,2,4-thiadiazole ring is essential for the reaction.

In this sense, theoretical data from the [tdzHMe_2_]^+^ and [tdzHMeN]^+^ are in nice agreement with the hypothesis reported in literature, where it is described that the thiolate form of cysteine-containing enzymes can attack the S atom from protonated thiadiazoles forming a disulfide bond with concomitant ring opening and to the enzyme inhibition [21, 22].

**Fig. 7:**
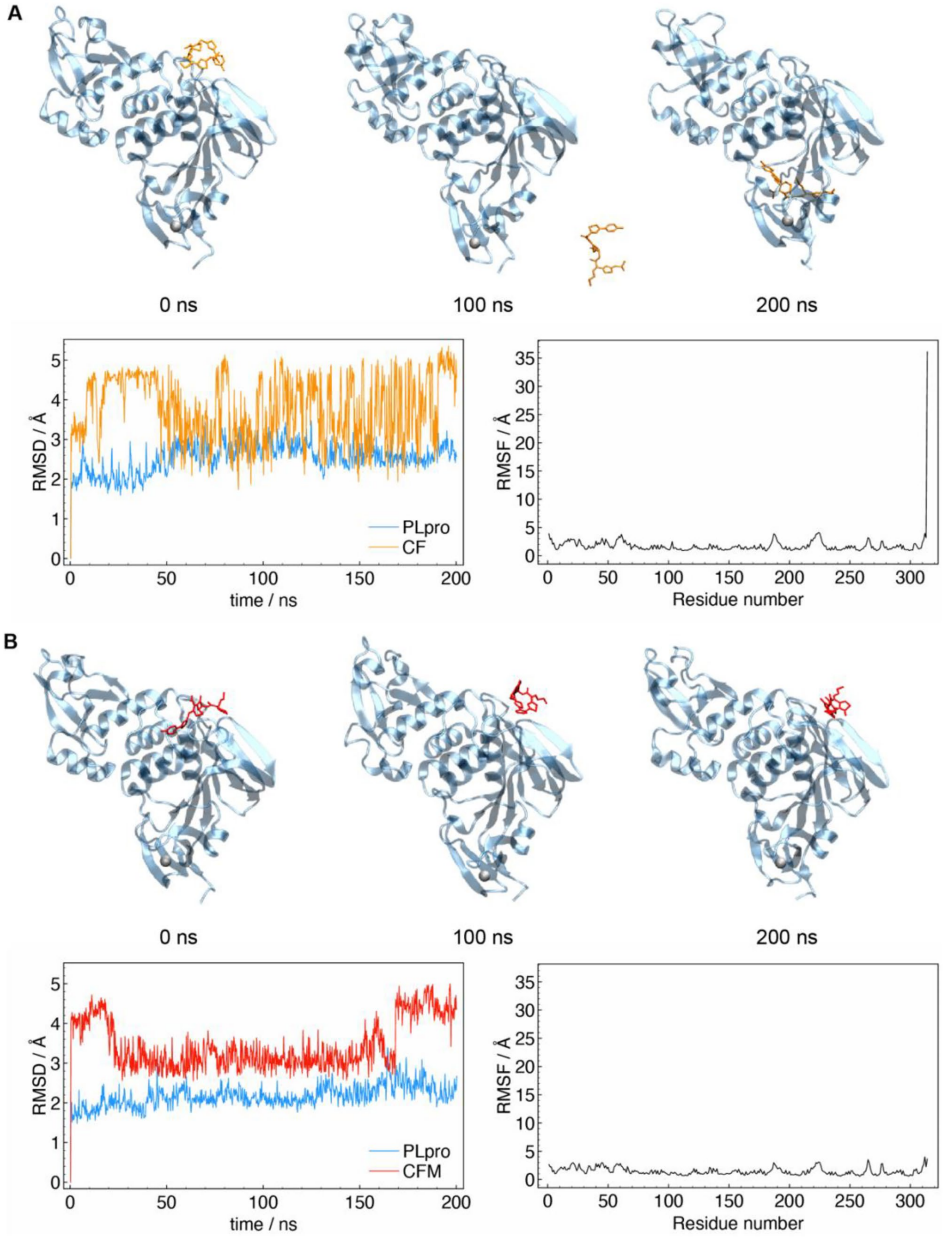
**A** MD structures of PL^pro^-ceftaroline fosamil at 0, 100, and 200 ns (top row), RMSD along the dynamics (bottom left), and RMSF by residue (bottom right); **B** MD structures of PL.^pro^- dephosphorylated ceftaroline fosamil metabolite at 0, 100, and 200 ns (top row), RMSD along the dynamics (bottom left), and RMSF by residue (bottom right)

### Molecular dynamics simulation of PL^pro^ with ceftaroline fosamil and metabolite isomer *Z*

Molecular dynamics (MD) simulations were performed on two selected systems, i.e., PL^pro^ with ceftaroline fosamil and dephosphorylated metabolite isomer *Z*, respectively. The results indicated two different scenarios. The calculations on the PL^pro^ complex with ceftaroline fosamil isomer *Z ***(**PL^pro^-ceftaroline fosamil) clearly indicated that this structure is possibly not an efficient inhibitor of the PL^pro^. After 200 ns of MD, the interactions between ceftaroline fosamil and the enzyme were not strong enough to maintain the antibiotic inside of PL^pro^ active site. In fact, already after 50 ns, the ligand was not bonded anymore to the protein structure, and it is moving freely in the simulation box. This was reflected from the very high RMSF attributed to the ceftaroline fosamil molecule, which was calculated to be 36.1 Å (**Fig.** **7****A**) (ceftaroline corresponds to the last residue in the RMSF graph). During the dynamics, there were two brief windows in which the ligand showed interaction with two different regions of the protein. From approximately 60 to 90 ns, it is found in proximity of Gln212 and Gln218 and from 190 ns until the end of the simulation around Leu122 as it can be seen from the RMSD graph (**Fig.** **7****A**) that showed lower fluctuations in these intervals. Longer simulations and further analyses will be required to assess if any of these sites could be relevant to the inhibitory action of ceftarolinefosamil (isomer *Z*).

Conversely, the MD simulations on the PL^pro^ ceftaroline fosamil dephosphorylated metabolite isomer *Z* show that this ligand tends to remain the same region during the entire simulation (see, for instance, the structures extracted at 100 and 200 ns in **Fig.** **7****B**). This reflected in the RMSD fluctuations which are noticeably lower than those of ceftaroline fosamil in PL^pro^. The RMSF value for ceftaroline fosamil dephosphorylated metabolite (3.6 Å) was found to be comparable to other residues in the protein. Interestingly, during the dynamics there are two sudden changes in conformation of ceftarolinefosamil metabolite at approximately 25 and 175 ns, which reflected in the abrupt changes in RMSD values. The structure, which is initially found to resemble that of a linear alkane, moved toward a more spherical geometry around 25 ns as the outermost parts bent inward toward the center of the molecule. This configuration is seen for approximately 150 ns after which another distention toward a more linear structure was seen. These three conformations (linear/spherical/linear) can be seen in **Fig.** **7****B** at 0, 100 and 200 ns, respectively. Nevertheless, during whole simulation dephosphorylated ceftaroline fosamil metabolite maintained its interaction with the initial binding site in the PL.^pro^. The effective interaction during the entire simulation may indicate that the dephosphorylated ceftaroline fosamil metabolite is a potential inhibitor of the enzyme.

## Conclusions

In this work, we have studied the mechanism of inhibition of 1,2,4-thiadiazole containing drugs (ceftaroline fosamil, ceftobiprole, and ceftobiprole medocaril, and their metabolites and isomers), combining molecular dynamics, docking, and quantum chemistry calculations. Our results suggest that the PL^pro^ enzyme may be a better target for these class of drugs than M^pro^, indicating that these compounds and metabolites should be tested in *in vitro/vivo* assays to confirm their pharmacological action. We verified that the *E* isomers of the studied drugs and metabolites have more favorable S···S interactions with the protease M^pro^. However, in the PL^pro^ protease, the *Z* isomer compounds showed the most favorable interactions, with the exception of the drug ceftobiprole medocaril. These results help the understanding of the interaction mode and the design of new compounds.

These conclusions are based on the interactions, binding poses, and S···S distances (from 1,2,4-thiadiazole to Cys111). Specifically, the sulfur atom in the thiadiazole ring from the ceftaroline fosamil isomers and its *Z* active metabolite showed an adequate interaction with the thiol group of Cys111 from PL^pro^ active site, as well the isomer metabolites of ceftobiprole and the isomer *E*1 of the experimental drug ceftobiprole medocaril. This suggests that the inhibition might be covalent. In fact, by DFT calculations, we demonstrated that the adduct formation can be ergetically favorable. In this way, the use of these cephalosporin drugs and their metabolites might be an option in the search for antivirals against COVID-19. In addition, the approved drugs ceftaroline fosamil and ceftobiprole are widely used in cases of pneumonia and respiratory tract infections [30–32], and perhaps they may be also effective against coronaviruses (in addition to pulmonar bacteria).

We also demonstrated that cephalosporin drugs and their metabolites, containing the 1,2,4-thiadiazole moiety, have a potential to be explored experimentally against COVID-19 and may bind to the SARS-CoV-2 M^pro^ and PL^pro^. According to MD and docking results, the dephosphorylated ceftaroline fosamil metabolites are able to interact with the Cys residues from and PL^pro^; those molecules might be a promising strategy to find new antivirals.

Overall, these data suggest that antibiotic drugs, as well as their derivatives containing the 1,2,4-thiadiazole ring, could be an option to repurposing drugs and as a perspective of this work, should be tested in *in vitro* and *in vivo* to confirm the inhibitory and pharmacological potential against the coronavirus. Thus, in addition to their antibacterial effects, ceftaroline fosamil, ceftobiprole, and ceftobiprole medocaril may be also effective inhibitors of the thiol-containing enzymes of the SARS-CoV-2.

## Supplementary Information

Below is the link to the electronic supplementary material.

Supplementary file1 (DOCX 9357 KB)

## Data Availability

Data and materials are freely accessible in the supporting information.
