# Supplementary material for: In silico studies of Mpro and PLpro from SARS-CoV-2 and a new class of cephalosporin drugs containing 1,2,4-thiadiazole
Source: Struct Chem. 2022 Sep 10;33(6):2205–20. doi: 10.1007/s11224-022-02036-5 (PMC9463509; doi:10.1007/s11224-022-02036-5)
Supplement: Supplementary file 1 — Supplementary file1 (DOCX 9357 KB) [file 11224_2022_2036_MOESM1_ESM.docx]

*Supporting information*

***In silico* studies of M^pro^ and PL^pro^ from SARS-CoV-2 and a new class of Cephalosporin drugs containing 1,2,4-thiadiazole**

Cássia Pereira Delgado^[a]^, João Batista Teixeira Rocha^[a]^, Laura Orian^[b]^, Marco Bortoli^[c]^ and Pablo Andrei Nogara^[a]*^

^a^Departamento de Bioquímica e Biologia Molecular, Universidade Federal de Santa Maria (UFSM), Santa Maria, 97105-900, RS, Brazil; *Corresponding author: [pbnogara@gmail.com](mailto:pbnogara@gmail.com).

^b^Dipartimento di Scuenze Chimiche, Università degli Studi di Padova, Via Marzolo 1 35131 Padova, Italy; *Corresponding author:[laura.orian@unipd.it](mailto:laura.orian@unipd.it)

^c^Institut de Química Computacionali Catàlisi (IQCC) Departament de Química, Facultat de Ciències, Universitat de Girona, C/M. A. Capmany 69,17003 Girona, Spain: [marco.bortoli@udg.edu](mailto:marco.bortoli@udg.edu)

**Summary**

|  |  |  |
| --- | --- | --- |
| Figure S1 | ……………………………………………………………………… | 2 |
| Table S1 | ……………………………………………………………………… | 2 |
| Figure S2 | ……………………………………………………………………… | 3 |
| Figure S3 | ……………………………………………………………………… | 3 |
| Figure S4  Table S2  Figure S5  Table S3  Figure S6 | ………………………………………………………………………  ………………………………………………………………………  ………………………………………………………………………  ………………………………………………………………………  ……………………………………………………………………… | 4  4  5  5  6 |
| Figure S7 | ……………………………………………………………………… | 6 |
| Figure S8 | ……………………………………………………………………… | 7 |
| Figure S9 | ……………………………………………………………………… | 8 |
| Figure S10  Figure S11  Figure S12  Figure S13 | ………………………………………………………………………  ………………………………………………………………………  ………………………………………………………………………  …………………………………………………………………… | 8  9  9  10 |
| Figure S14 | ……………………………………………………………………… | 11 |
| Figure S15 | ……………………………………………………………………… | 12 |
| Figure S16 | ……………………………………………………………………… | 13 |
| Table S4 | ……………………………………………………………………… | 14 |

**
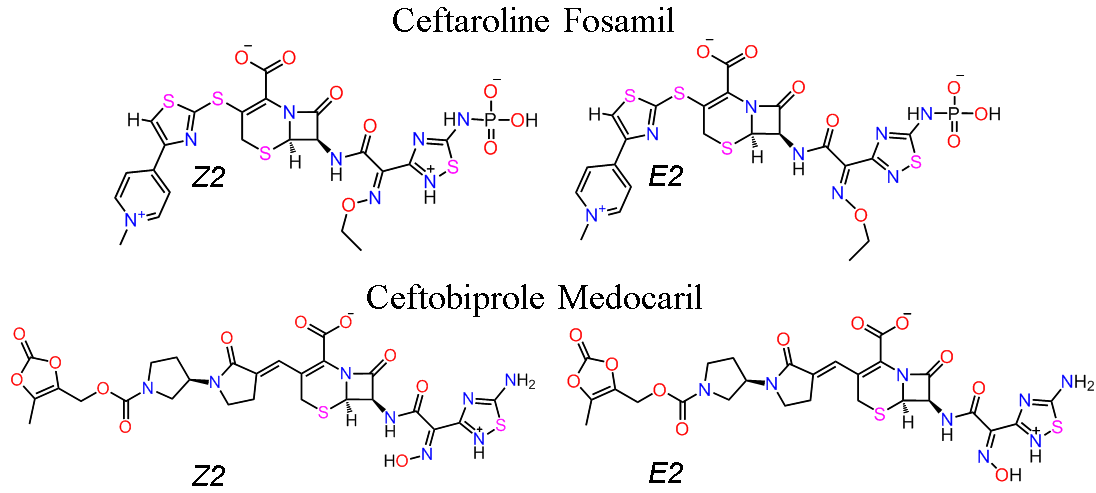
Figure S1**. Chemical structure of ceftaroline fosamil and ceftobiprole medocaril isomers (*Z, E*)2 drugs, approved and experimental, respectively. The lowest populous isomers, according to the Marvin Skecth simulations.

**Table S1**. Marvin Sketch pKa simulations with 1,2,4-thiadiazoles drugs in the physiological pH 7.0 -7.4. The (*Z,E*)1 and (*Z,E*)2 isomers showed the percentage (%) of etch pH status.

| **Molecule (*Z,E*)1** | pH 7,0 (%) | pH 7,2 (%) | pH 7,4 (%) | **Molecule (*Z,E*)2** | pH 7  (%) | pH 7,2 (%) | pH 7,4 (%) |
| --- | --- | --- | --- | --- | --- | --- | --- |
| ceftaroline fosamil | 60.50 | 70.82 | 79.34 | ceftaroline fosamil | 39.36 | 29.06 | 20.55 |
| ceftobiprole medocaril | 93.31 | 95.67 | 97.22 | ceftobiprole medocaril | 6.67 | 4.31 | 2.76 |

**
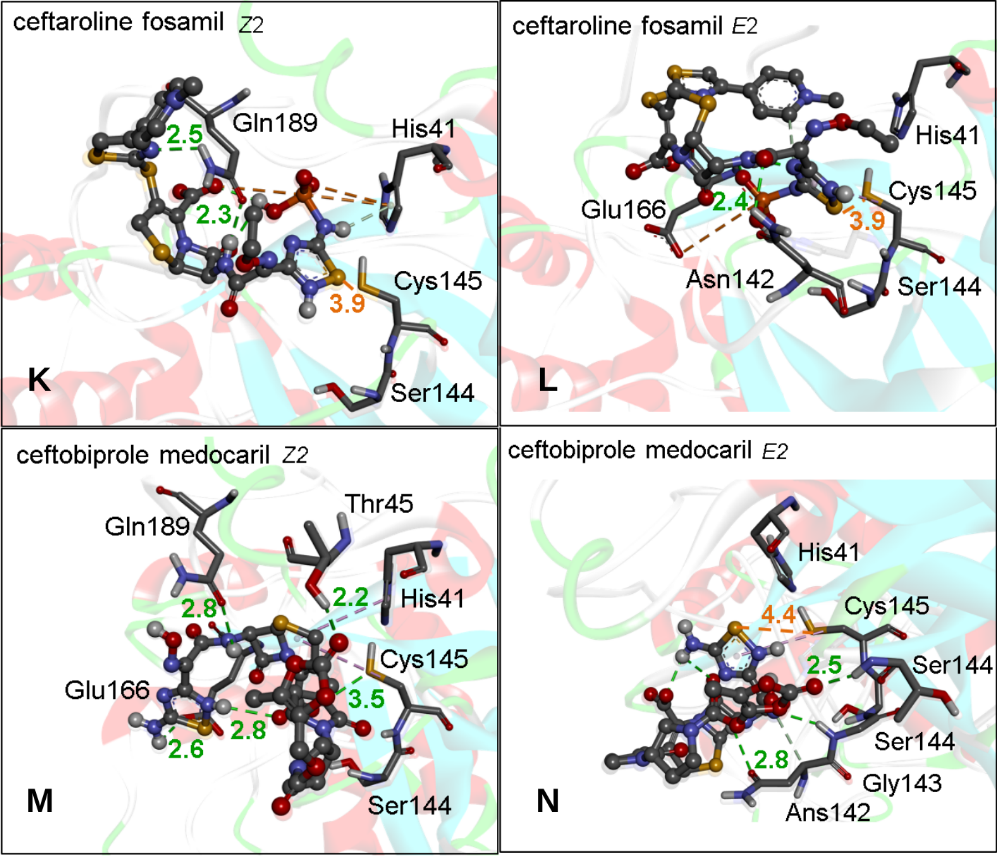
**

**Figure S2**. M^pro^ docking with 1,2,4-thiadiazole drugs in the (*Z,E*)2 isomers in the best distance conformer. **K**) ceftaroline fosamil isomer *Z2*. **L**) ceftaroline fosamil isomer *E2*. **M**) ceftobiprole medocaril Z2. **N**) ceftobiprole medocaril *E*2. Distances are shown in Å.

**
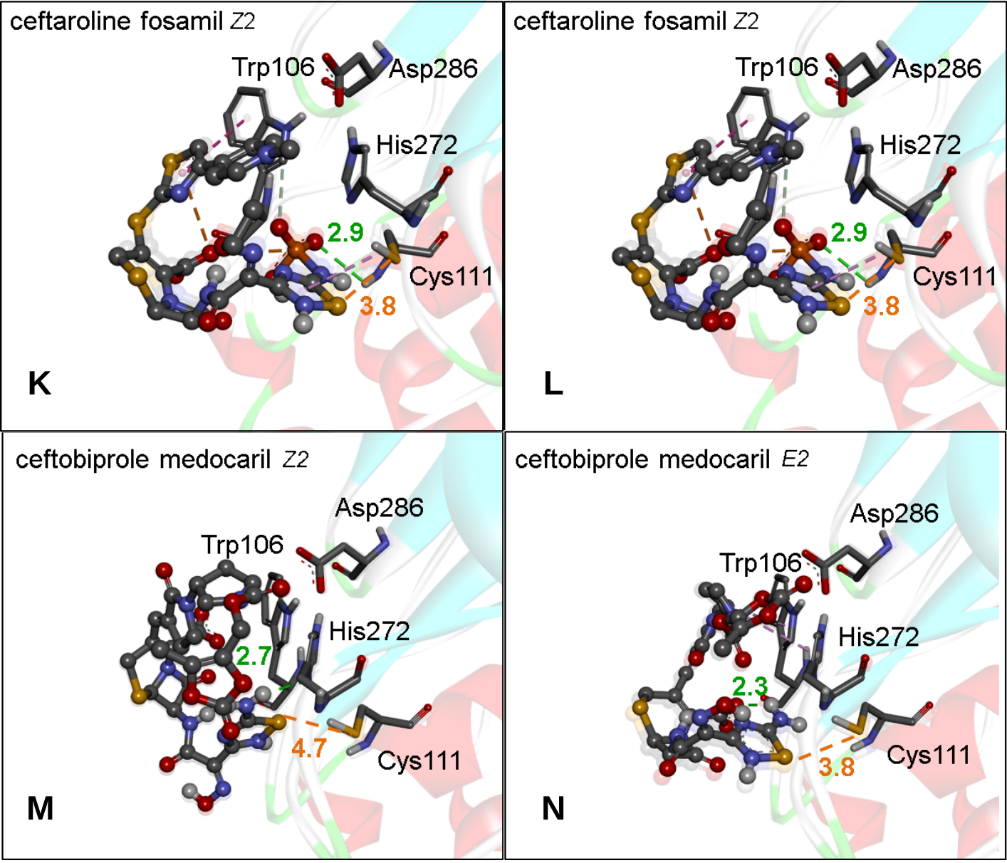
**

**Figure S3**. PL^pro^ docking with 1,2,4-thiadiazole drugs in the (*Z,E*)2 isomers in the best distance conformer. **K**) ceftaroline fosamil isomer *Z2*. **L**) ceftaroline fosamil isomer *E2*. **M**) ceftobiprole medocaril Z2. **N**) ceftobiprole medocaril *E*2. Distances are shown in Å.

**
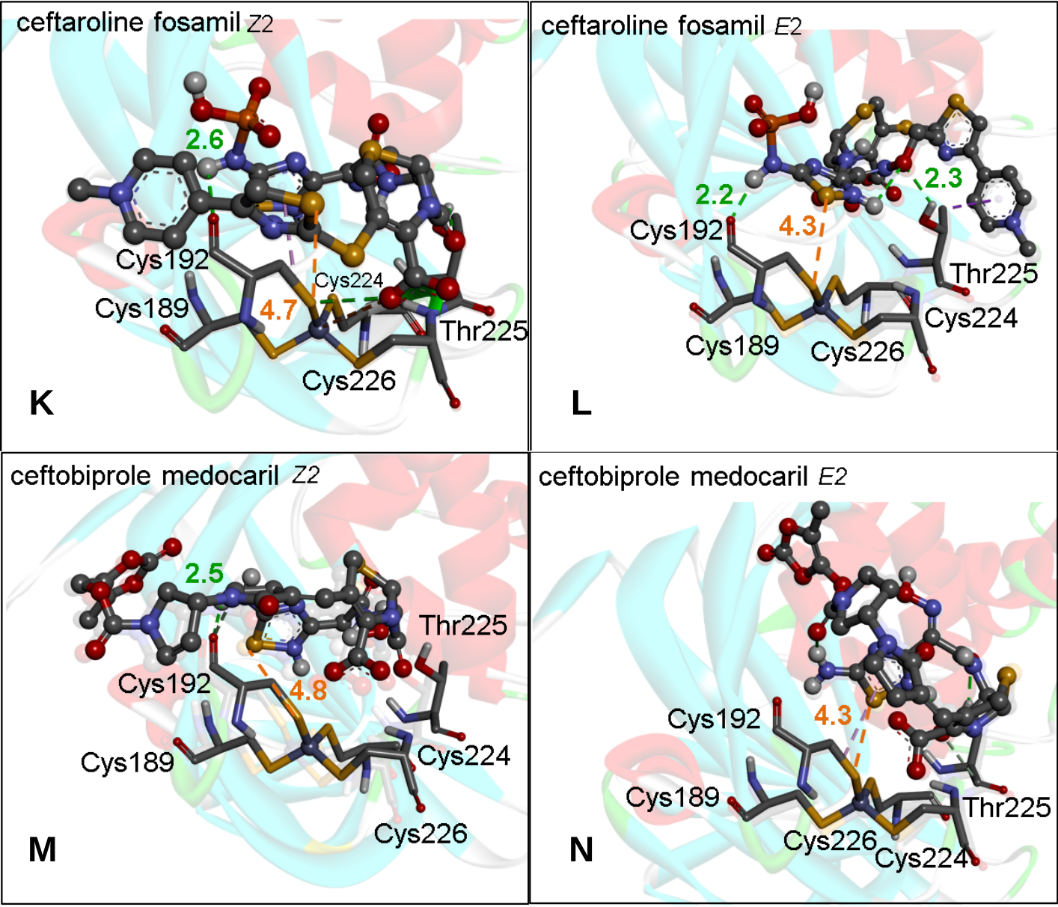
**

**Figure S4**.PL^pro^ Zn binding site docking with 1,2,4-thiadiazole drugs in the (*Z,E*)2 isomers in the best distance conformer. . **K**) ceftaroline fosamil isomer *Z2*. **L**) ceftaroline fosamil isomer *E2*. **M**) ceftobiprole medocaril Z2. **N**) ceftobiprole medocaril *E*2. Distances are shown in Å.

**Table S2**. Predicted binding free energies (∆G, kcal∙mol^-1^) between Mpro and PLpro and PLpro Zn biding site, with 1,2,4-thiadiazoles drugs (Z, E)2 isomers, Ceftaroline Fosamil and Ceftobiprole Medocaril, respectively.

|  | **^a^M^pro^** |  | **^b^PL^pro^** |  | **^c^PL^pro^** | **Zn** |
| --- | --- | --- | --- | --- | --- | --- |
| Molecule | **∆G** | dist. (Å)  S*∙∙∙S (Cys145) | **∆G** | dist. (Å)  S*∙∙∙S (Cys111) | **∆G** | dist. (Å)  S*∙∙∙S (Cys192) |
| Ceftaroline Fosamil *Z2* | -7.4 | 3.9 | -5.7 | 3.8 | -4.7 | 4.7 |
| Ceftaroline Fosamil *E2* | -7.0 | 3.9 | -5.8 | 3.4 | -5.4 | 4.3 |
| Ceftobiprole Medocaril *Z2* | -7.2 | - | -6.1 | 4.7 | -5.5 | 4.8 |
| Ceftobiprole Medocaril *E2* | -7.0 | 4.4 | -5.9 | 3.8 | -5.6 | 4.3 |

Interact: electrophile center of the ligand, S*∙∙∙S of the 1,2,4 thiadiazole heterocycle; Distance (in Å) of the thiol from ^a^Cys145, ^b^Cys111, and ^c^Cys192 to the ligand; Green, yellow, and red colors indicate a favorable, intermediate, and less favorable interaction, respectively.


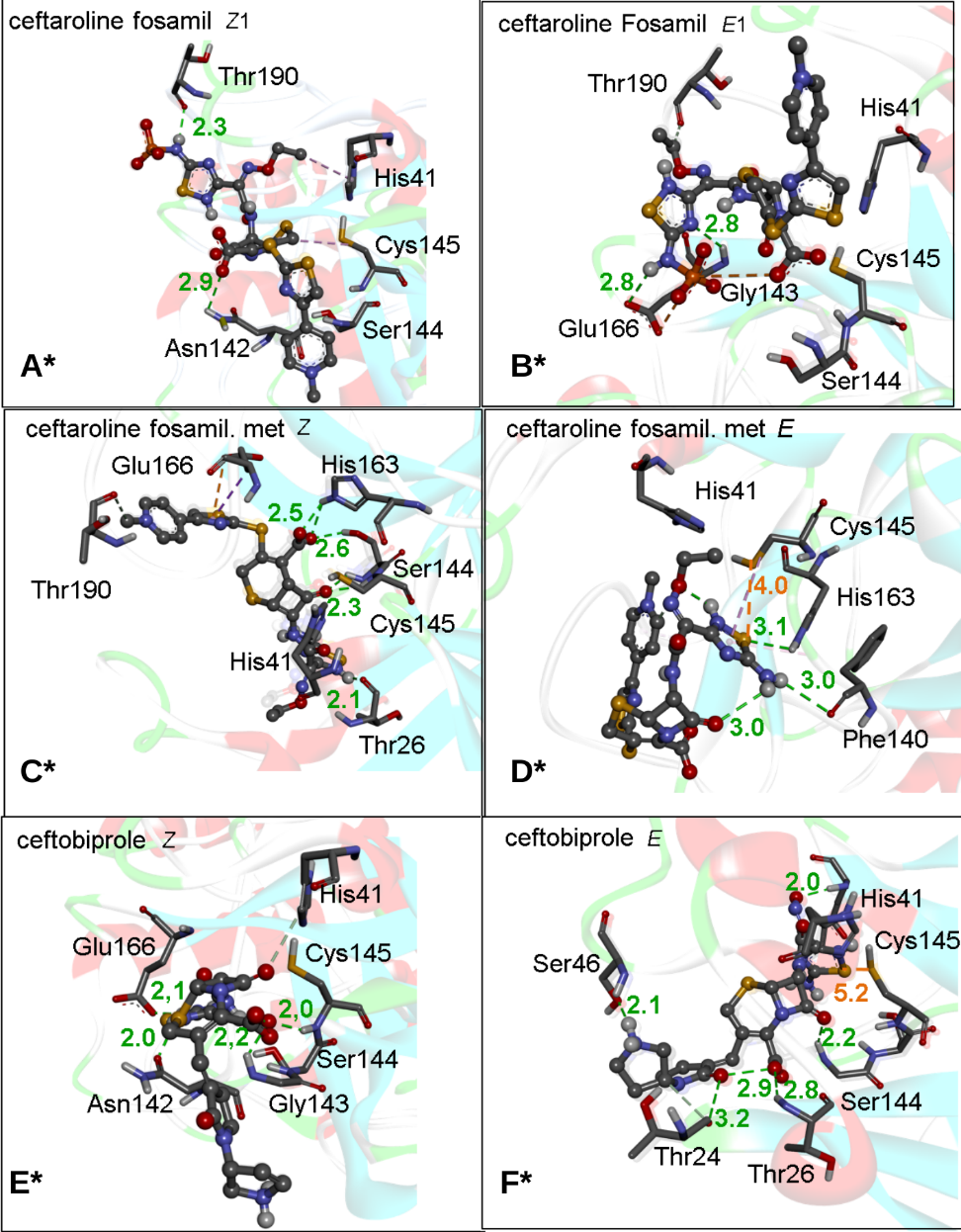


**Figure S5**. M^pro^ docking with 1,2,4-thiadiazole containing drugs and their metabolites in the larger negative binding energyconformer. **A**) ceftaroline fosamil isomer *Z1*. **B**) ceftaroline fosamil isomer *E1*. **C**) ceftaroline fosamil dephosphorylated metabolite isomer *Z*. **D**) ceftaroline fosamil dephosphorylated metabolite isomer *E*. **E**) ceftobiprole isomer *Z*. **F**) ceftobiprole isomer E. Distances are shown in Å.

**Table S3**. Predicted binding free energies (∆G, kcal∙mol^-1^) between Mpro and PLpro and PLpro Zn biding site, with 1,2,4-thiadiazoles drugs (Z, E)2 isomers in the best conformer, Ceftaroline Fosamil and Ceftobiprole Medocaril, respectively.

|  | | **^a^M^pro^** | |  | | **^b^PL^pro^** | |  | | **^c^PL^pro^** | | **Zn** |
| --- | --- | --- | --- | --- | --- | --- | --- | --- | --- | --- | --- | --- |
| Molecule | | **∆G** | | dist. (Å) | | **∆G** | | dist. (Å) | | **∆G** | | dist. (Å) |
|  |  |  |  | S*∙∙∙S | |  |  | S*∙∙∙S | |  |  | S*∙∙∙S |
|  |  |  |  | (Cys145) | |  |  | (Cys111) | |  |  | (Cys192) |
| Ceftaroline Fosamil *Z2* | | -7.5 | | - | | -6.9 | | - | | -4.9 | | 5.0 |
| Ceftaroline Fosamil *E2* | | -7.3 | | 3,9 | | -6.4 | | - | | -5.8 | | 4.9 |
| Ceftobiprole Medocaril *Z2* | | -7.7 | | - | | -6.4 | | - | | -5.9 | | - |
| Ceftobiprole Medocaril *E2* | | -7.2 | | - | | -6.3 | | - | | -6.0 | |  |
|  |  | |  | |  | |  | |  | |  | |

Interact: electrophile center of the ligand, S*∙∙∙S of the 1,2,4 thiadiazole heterocycle; Distance (in Å) of the thiol from ^a^Cys145, ^b^Cys111, and ^c^Cys192 to the ligand; Green, yellow, and red colors indicate a favorable, intermediate, and less favorable interaction, respectively.


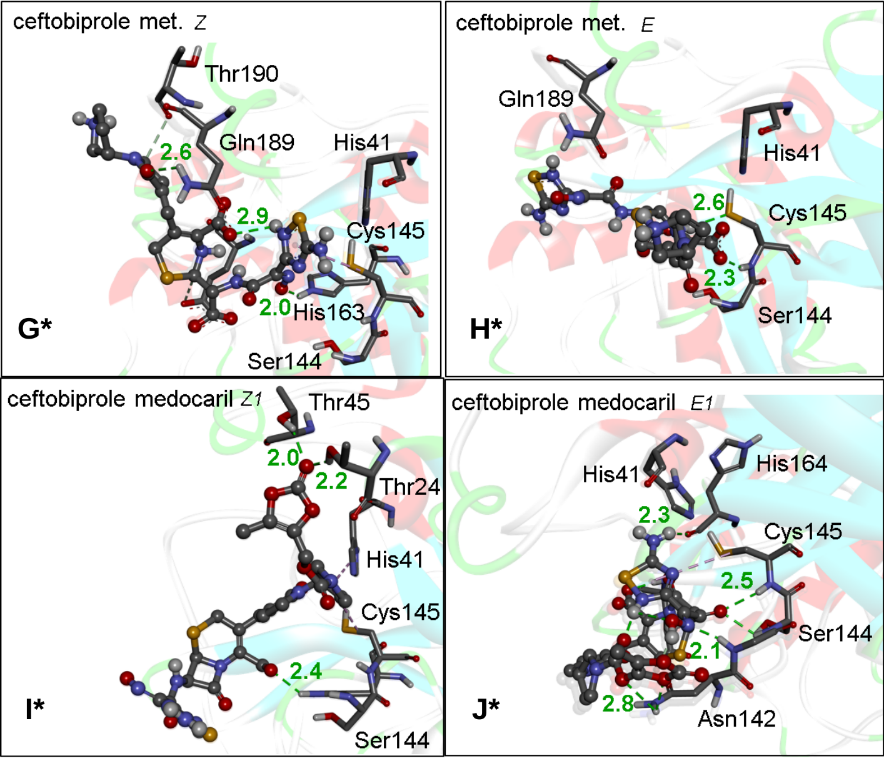


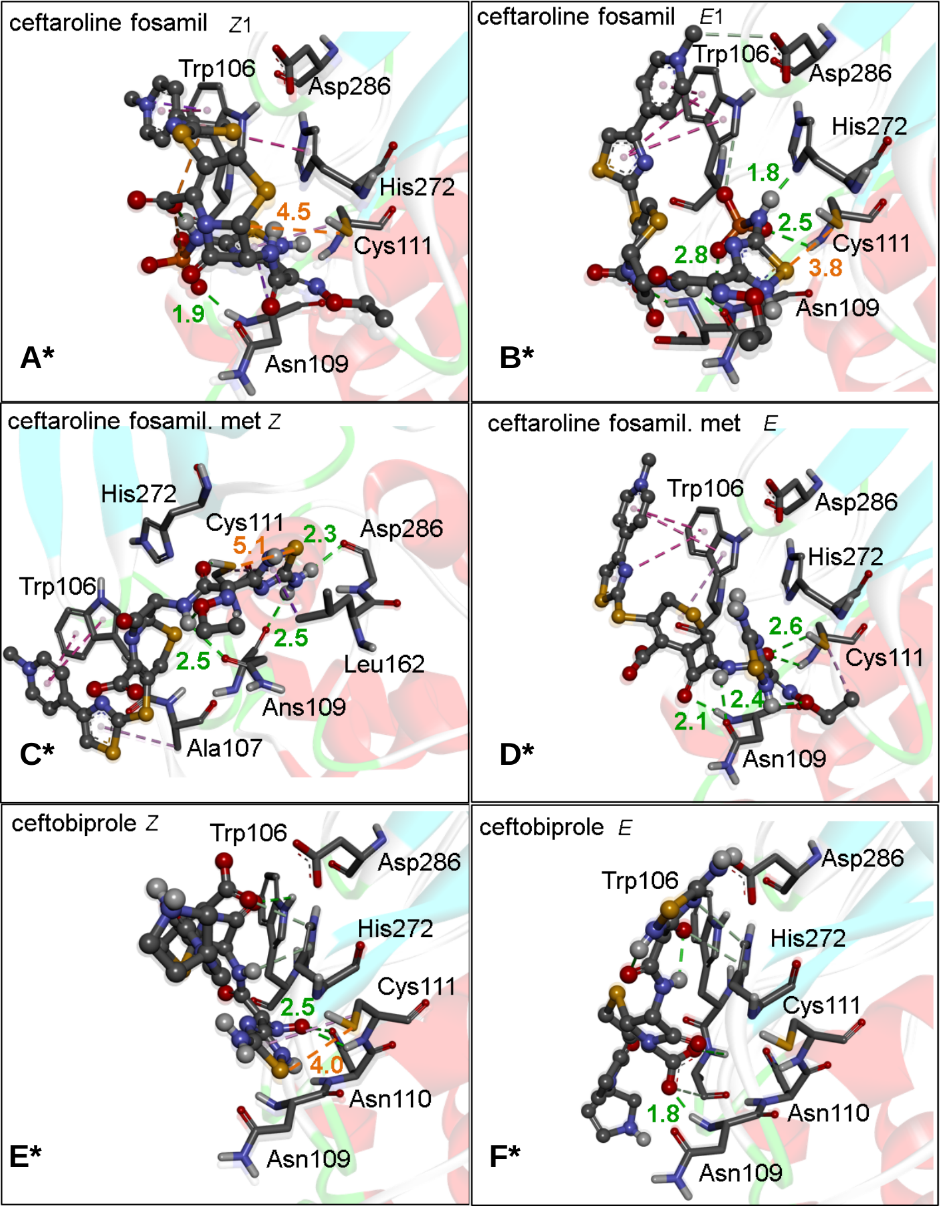
**Figure S6**. M^pro^ docking with 1,2,4-thiadiazole containing drugs and their metabolites in the larger negative binding energyconformer. **G**) ceftobiprole metabolite isomer *Z*. **H**) ceftobiprole metabolite isomer *E*. **I**) ceftobiprole medocaril isomer *Z1*.**J**) ceftobiprole medocaril isomer *E1*. Distances are shown in Å.

**Figure S7**. PL^pro^ docking with 1,2,4-thiadiazole drugs and its metabolites in the larger negative binding energyconformer. **A**) ceftaroline fosamil isomer *Z1*. **B**) ceftaroline fosamil isomer *E1*. **C**) ceftaroline fosamil dephosphorylated metabolite isomer *Z*. **D**) ceftaroline fosamil dephosphorylated metabolite isomer *E*. **E**) ceftobiprole isomer *Z*. **F**) ceftobiprole isomer E. Distances are shown in Å.

**
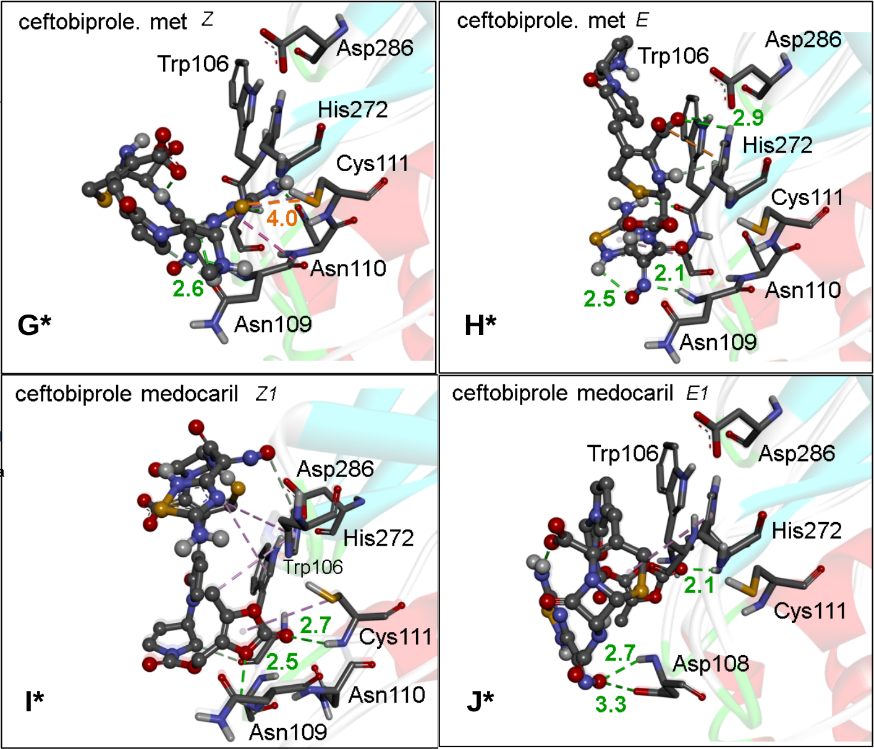
**

**Figure S8**. PL^pro^ docking with 1,2,4-thiadiazole drugs and its metabolites in the larger negative binding energyconformer. **G**) ceftobiprole metabolite isomer *Z*. **H**) ceftobiprole metabolite isomer *E*. **I**) ceftobiprole medocaril isomer *Z1*.**J**) ceftobiprole medocaril isomer *E1*. Distances are shown in Å.

**
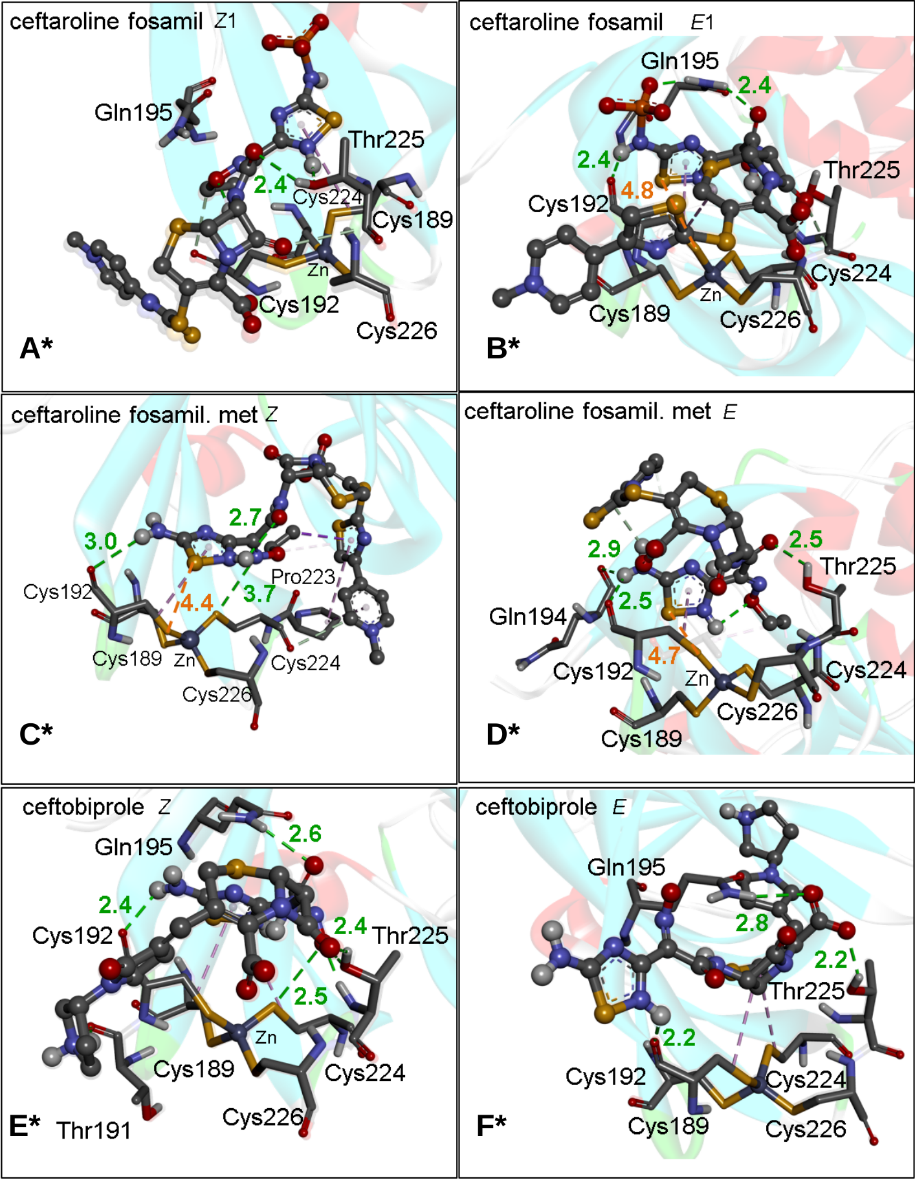
**

**
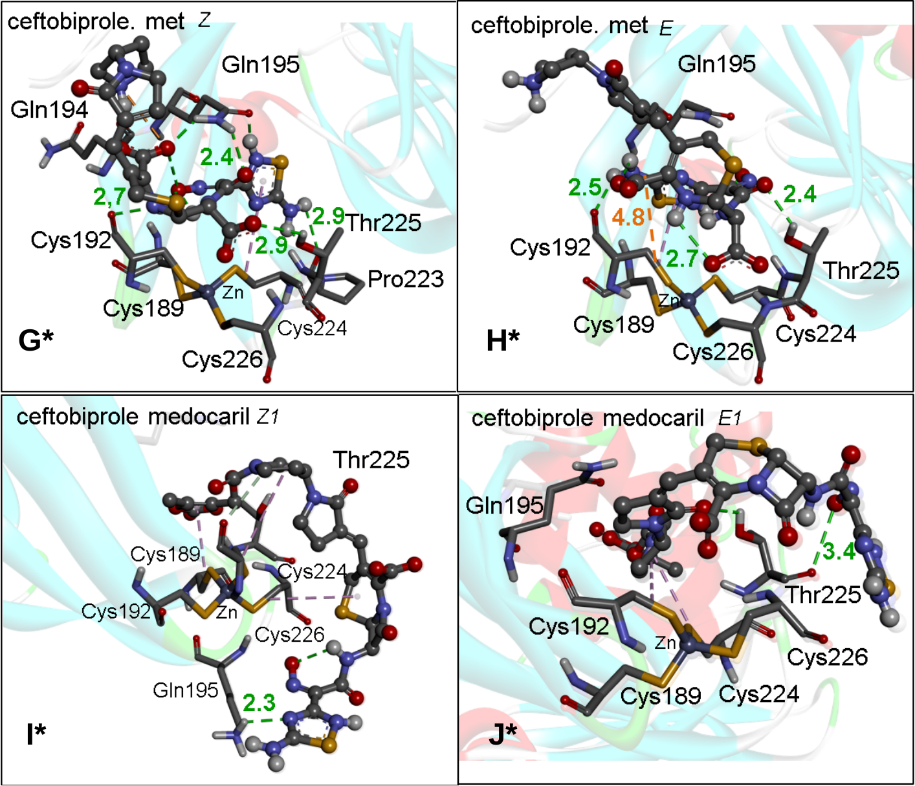
Figure S9**. PL^pro^Zn site docking with 1,2,4-thiadiazole drugs and its metabolites in the best energy conformer. **A**) ceftaroline fosamil isomer *Z1*. **B**) ceftaroline fosamil isomer *E1*. **C**) ceftaroline fosamil dephosphorylated metabolite isomer *Z*. **D**) ceftaroline fosamil dephosphorylated metabolite isomer *E*. **E**) ceftobiprole isomer *Z*. **F**) ceftobiprole isomer E. Distances are shown in Å.

**
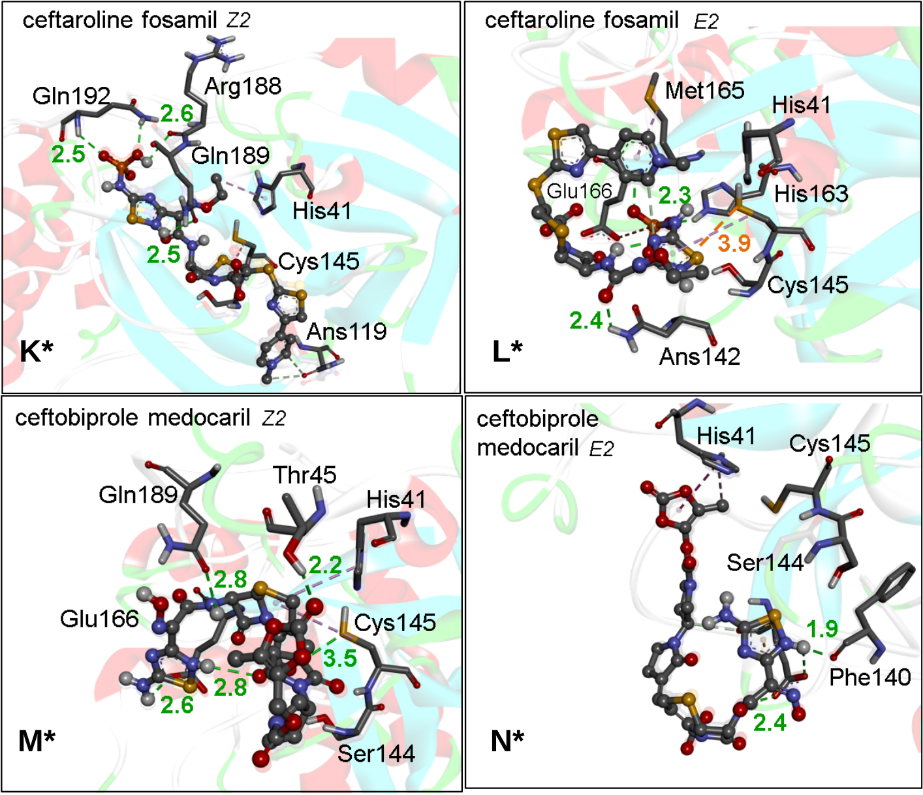
Figure S10**. PL^pro^ Zn binding site docking with 1,2,4-thiadiazole drugs and its metabolites in the larger negative binding energyconformer. **G**) ceftobiprole metabolite isomer *Z*. **H**) ceftobiprole metabolite isomer *E*. **I**) ceftobiprole medocaril isomer *Z1*.**J**) ceftobiprole medocaril isomer *E1*. Distances are shown in Å.

**Figure S11.** M^pro^ docking with 1,2,4-thiadiazole drugs in the (*Z,E*)2 isomers in the larger negative binding energyconformer. **K***) ceftaroline fosamil isomer *Z2*. **L***) ceftaroline fosamil isomer *E2*. **M***) ceftobiprole medocaril Z2. **N***) ceftobiprole medocaril *E*2. Distances are shown in Å.

**
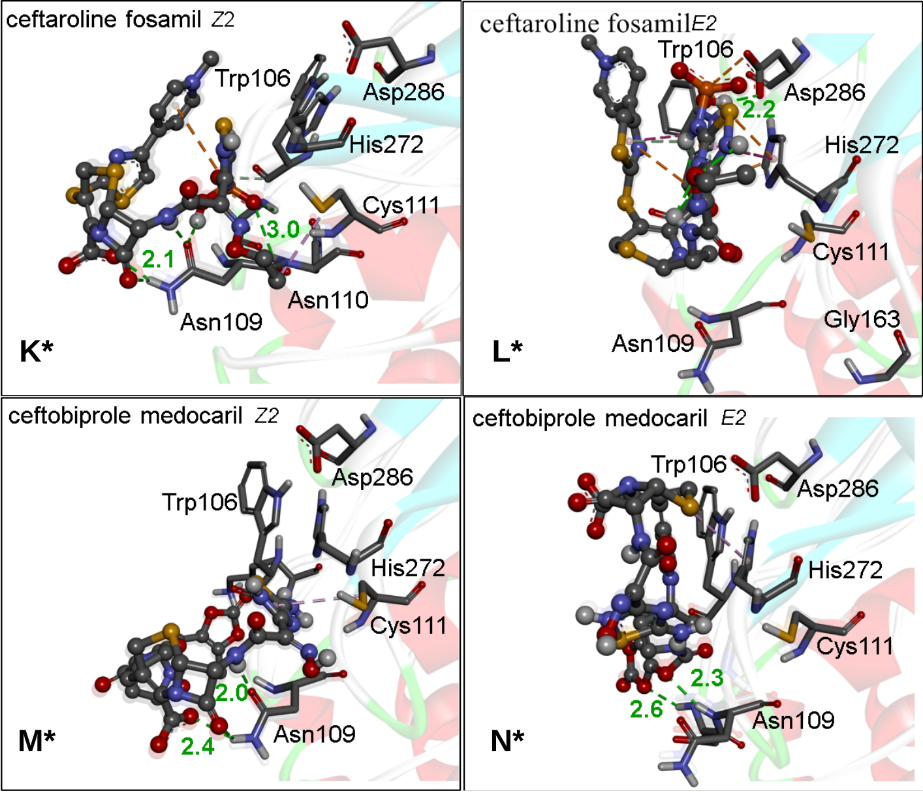
**

**Figure S12**. PL^pro^ docking with 1,2,4-thiadiazole drugs in the (*Z,E*)2 isomers in the larger negative binding energyconformer. **K***) ceftaroline fosamil isomer *Z2*. **L***) ceftaroline fosamil isomer *E2*. **M***) ceftobiprole medocaril Z2. **N***) ceftobiprole medocaril *E*2. Distances are shown in Å.

**
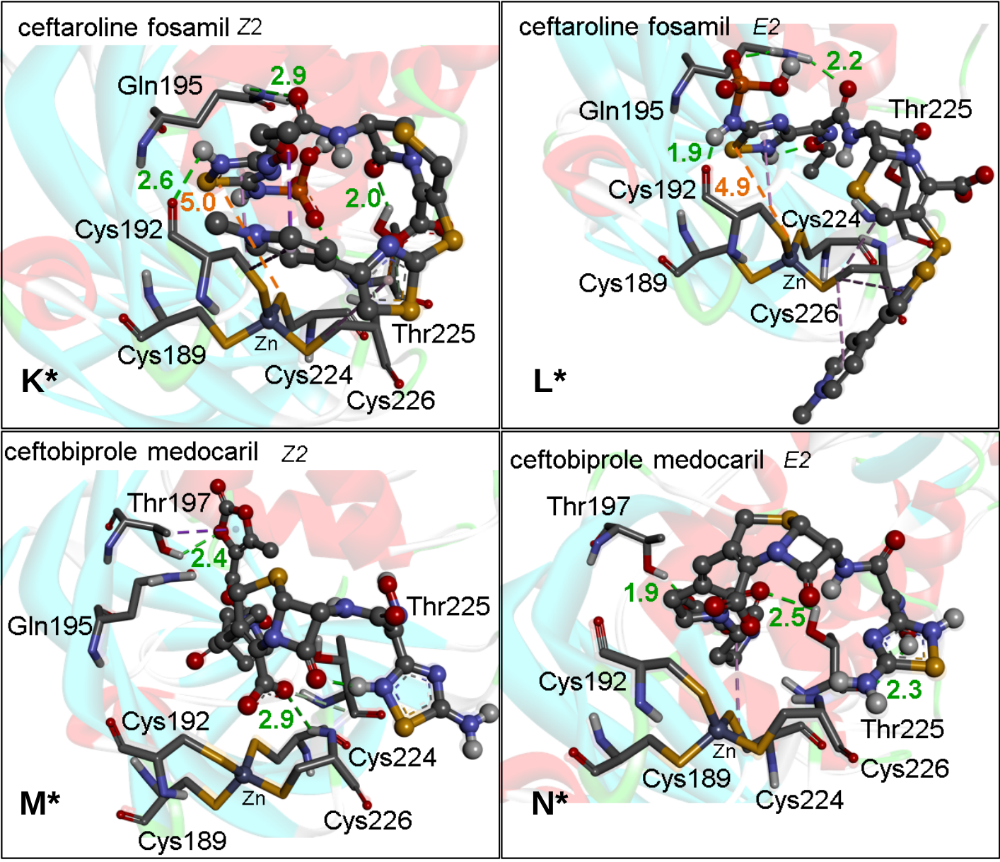
**

**Figure S13**. PL^pro^ Zn biding site docking with 1,2,4-thiadiazole drugs in the (*Z,E*)2 isomers in the larger negative binding energyconformer. **K***) ceftaroline fosamil isomer *Z2*. **L***) ceftaroline fosamil isomer *E2*. **M***) ceftobiprole medocaril Z2. **N***) ceftobiprole medocaril *E*2. Distances are shown in Å.

**
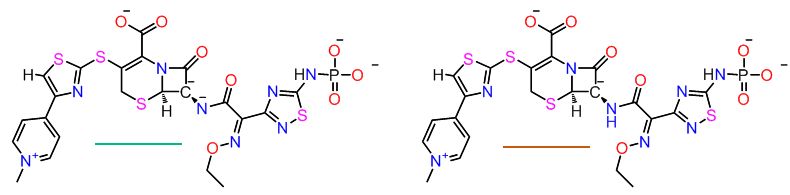

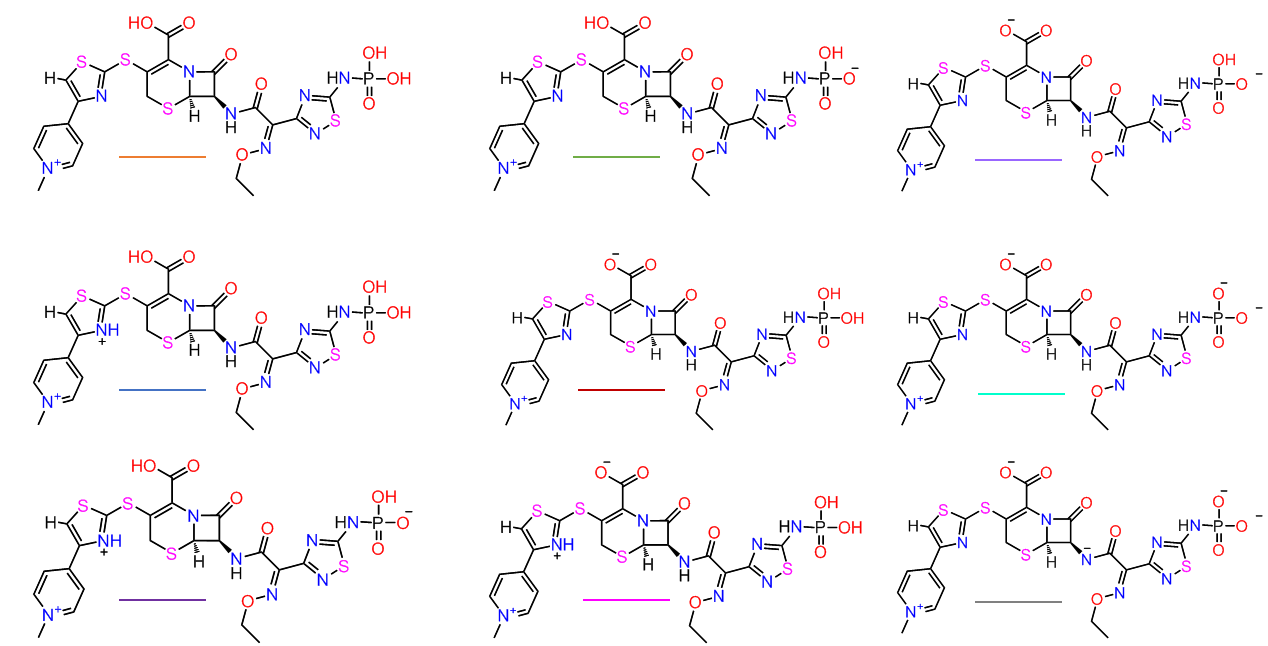

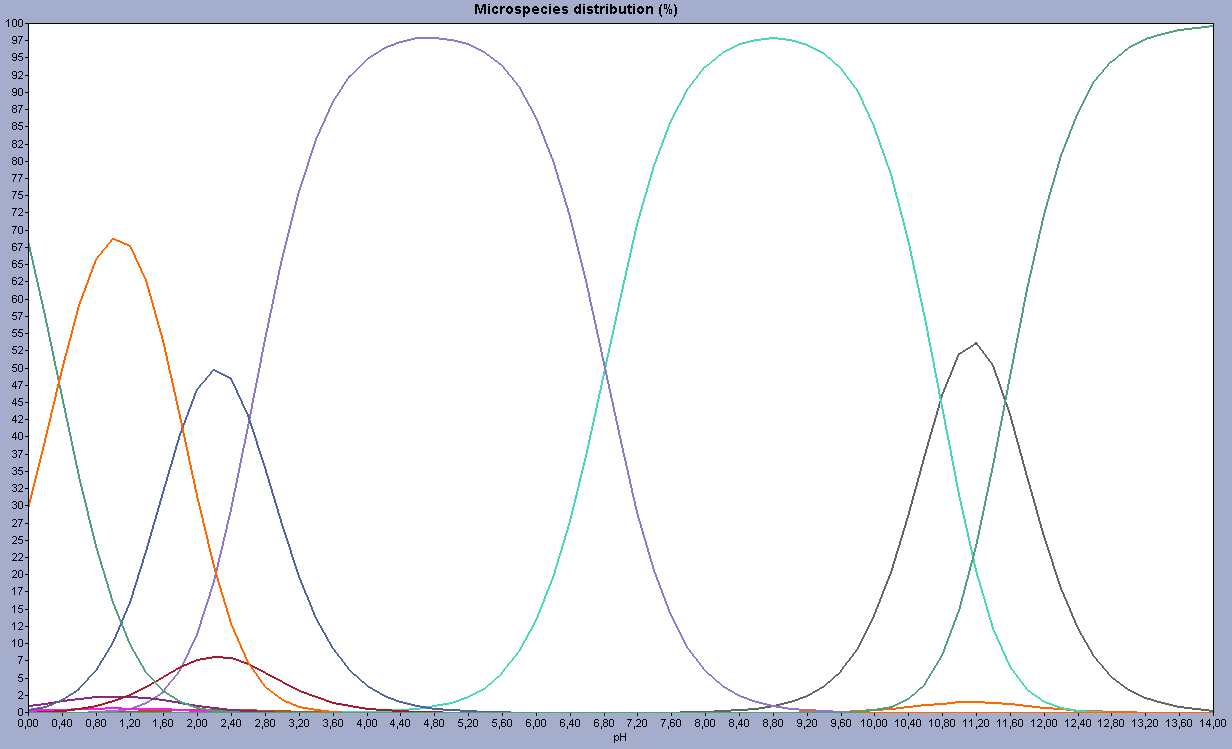
**

**Figure S14**. Marvin Sketch pKa simulations with Ceftaroline Fosamil approved drug in the pH between 0,0 -14. The pH equilibrium are show in %.


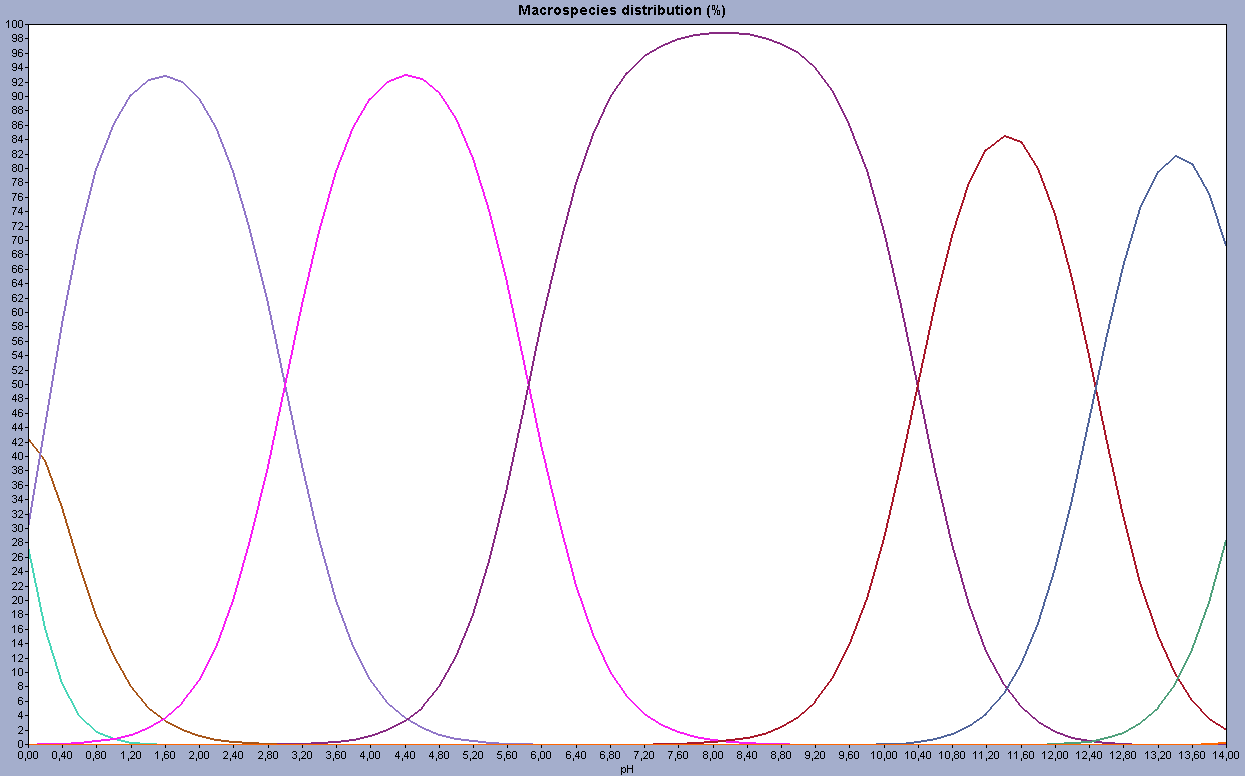


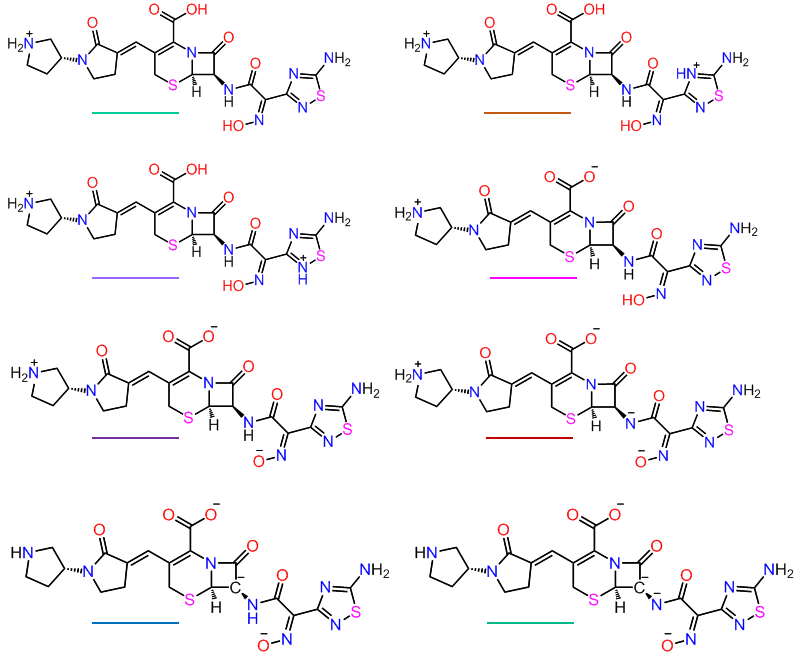


**Figure S15**. Marvin Sketch pKa simulations with Ceftobiprole approved drug in the pH between 0,0 -14. The pH equilibrium is show in %.


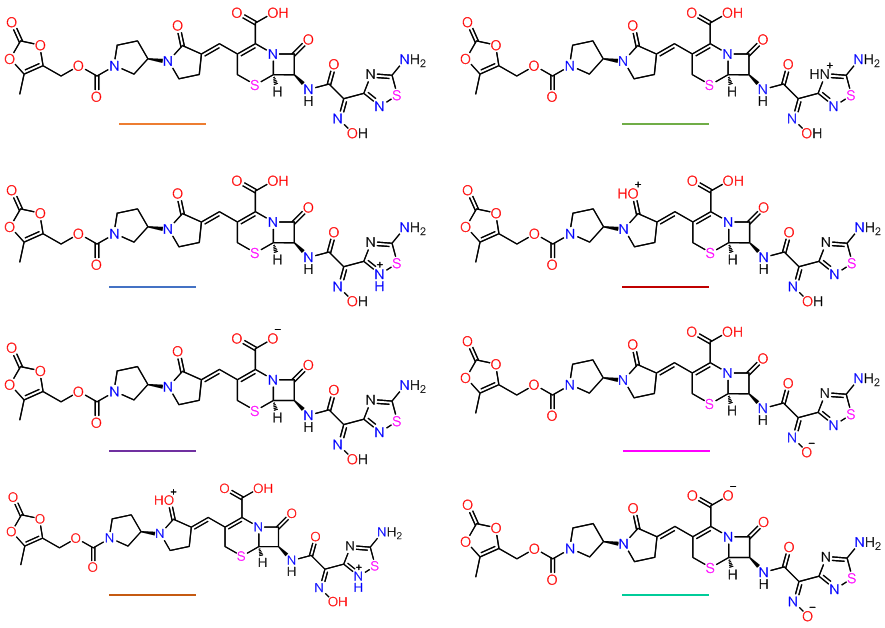
**
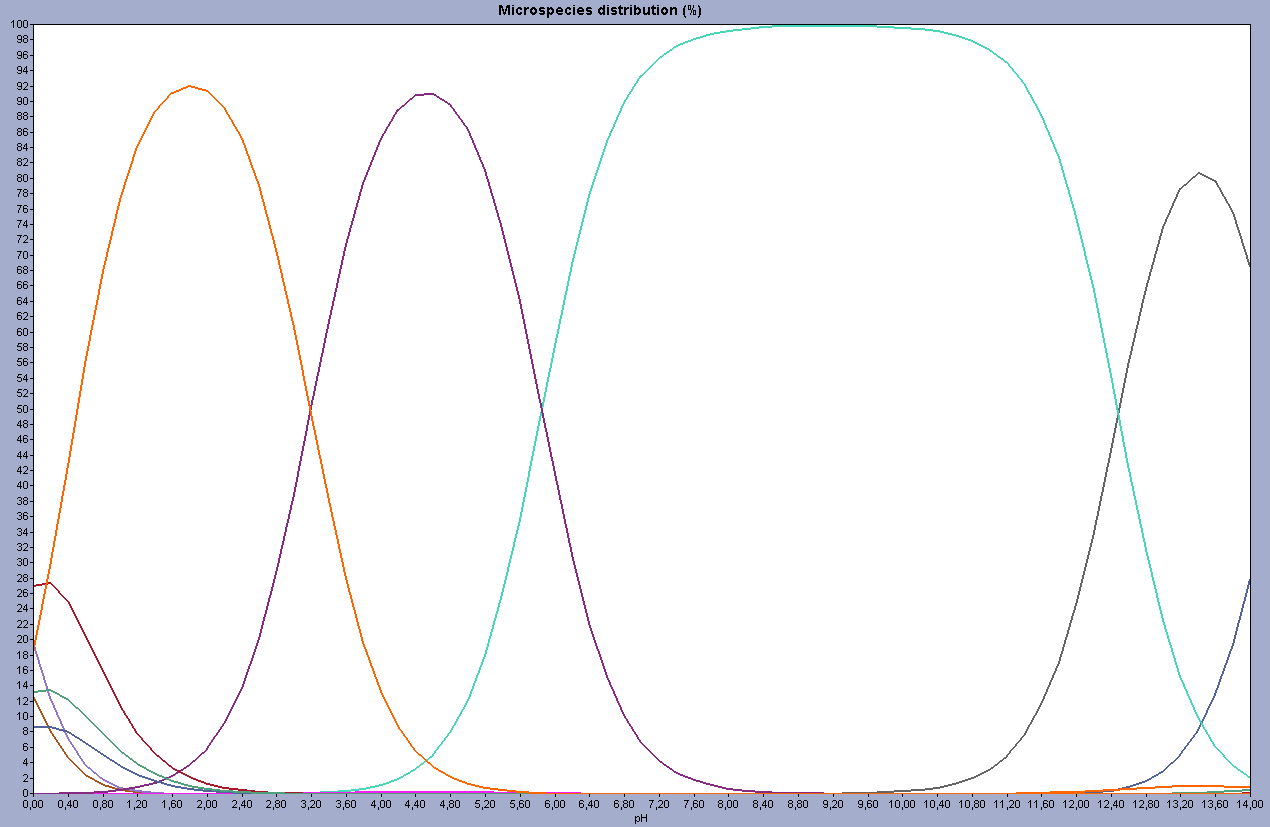
**
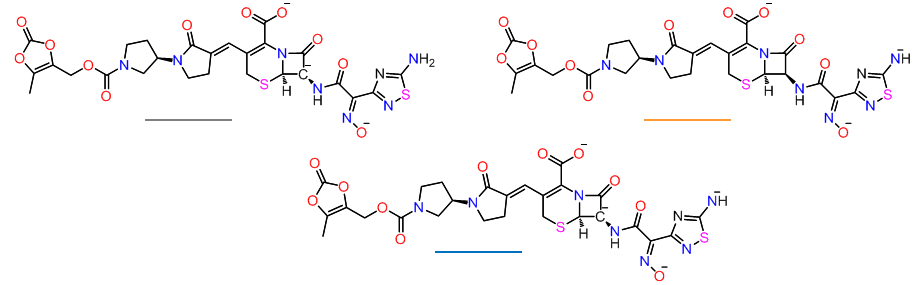


**Figure S16**. Marvin Sketch pKa simulations with Ceftobiprole Medocaril experimental drug in the pH between 0,0 -14. The pH equilibrium is show in %.

**Table S4.** Coordinates and energies (Ha) of optimized molecules. Level of theory ZORA-OLYP/TZ2P.

**MeS^-^** (gasphase)

E = -0.86001875

S -1.297868 0.612914 0.055954

C -0.892902 0.526119 -1.727900

H 0.129454 0.152005 -1.906805

H -0.957126 1.511562 -2.219835

H -1.573599 -0.144760 -2.279064

**[tdzHMe_2_]^+^** (gasphase)

E = -2.67337609

S -7.194778 1.851047 0.000000

C -7.639024 3.523754 0.000000

N -8.944108 3.713288 0.000000

C -9.621674 2.558941 0.000000

N -8.828722 1.472602 0.000000

H -9.147080 0.507689 0.000000

C -11.102983 2.475201 0.000000

H -11.465112 1.446320 0.000000

H -11.489828 2.999399 -0.880794

H -11.489828 2.999399 0.880794

C -6.625944 4.607810 0.000000

H -5.602792 4.228901 0.000000

H -6.779863 5.239810 0.882139

H -6.779863 5.239810 -0.882139

**tdzHMe_2_-SMe** (gasphase)

E = -3.74126805

S -3.324099 1.955655 -0.570193

C -3.954585 0.499217 -1.463871

N -5.203687 0.345715 -1.627054

C -6.231869 1.189564 -1.186139

N -6.440364 2.303715 -1.780551

H -7.284184 2.740155 -1.404189

C -7.076188 0.556885 -0.099566

H -7.890710 1.219964 0.199098

H -6.462964 0.329086 0.779339

H -7.495559 -0.390245 -0.454765

C -2.984527 -0.487619 -2.046542

H -2.382011 -0.965896 -1.269692

H -2.288450 0.011414 -2.727223

H -3.548178 -1.248094 -2.588515

S -1.358792 1.634817 -0.110304

C -1.396913 0.788227 1.502654

H -0.349425 0.713258 1.812646

H -1.821074 -0.214926 1.421290

H -1.949233 1.378041 2.236577

**Cys^-^** (gasphase)

E = -2.70512220

S -1.409996 1.852500 -0.119407

C -1.203072 0.881588 1.407861

C 0.283778 0.765301 1.881818

N 1.085712 1.955478 1.542086

H 0.516229 2.411443 0.788621

H 1.121649 2.594849 2.330703

H 0.283705 0.618287 2.971587

C 1.020461 -0.481488 1.337705

O 0.621429 -1.624526 1.452303

O 2.207185 -0.174618 0.779414

H 2.203455 0.830215 0.892826

H -1.589537 -0.136177 1.293854

H -1.766798 1.348148 2.231730

**tdzHMe_2_-Cys** (gasphase)

E = -5.55371882

S -2.894904 1.518082 -0.956163

C -4.026396 0.229550 -1.577470

N -5.282459 0.393969 -1.499659

C -5.970143 1.497252 -0.978679

N -5.919373 2.625838 -1.580809

H -6.558176 3.285007 -1.131649

C -6.828961 1.131776 0.213364

H -7.373423 2.001679 0.586206

H -6.211061 0.725102 1.021808

H -7.544662 0.351723 -0.066365

C -3.457025 -1.001150 -2.220657

H -2.869780 -1.589121 -1.509950

H -2.787578 -0.735267 -3.043935

H -4.282284 -1.607057 -2.596633

S -1.024364 0.699379 -0.817768

C -0.929778 -0.011255 0.883701

C 0.154070 0.641276 1.757686

N 0.082008 2.093546 1.930920

H -0.250449 2.531752 1.074720

H -0.557247 2.350203 2.675363

H 0.056789 0.185295 2.753255

C 1.588919 0.231338 1.313997

O 1.867326 -0.871135 0.905744

O 2.495276 1.206925 1.487370

H 1.959769 1.975336 1.804946

H -0.716074 -1.077631 0.782855

H -1.913195 0.118104 1.339038

**tdzMe_2_** (gasphase)

E = -2.78504704

S -3.315506 0.843079 0.000000

C -3.677716 2.533602 0.000000

N -4.974769 2.730881 0.000000

C -5.624479 1.523186 0.000000

N -4.910143 0.407797 0.000000

C -7.121165 1.475473 0.000000

H -7.469089 0.442184 0.000000

H -7.516740 1.991044 -0.881468

H -7.516740 1.991044 0.881468

C -2.652842 3.621032 0.000000

H -1.635770 3.223007 0.000000

H -2.781137 4.255855 0.882996

H -2.781137 4.255855 -0.882996

**[tdzMe_2_-SMe]^-^** (TCI) (gas phase)

E = -3.66468410

S -3.760106 1.494944 -0.621478

C -3.899653 2.994310 0.245204

N -5.149672 3.293573 0.545542

C -5.973253 2.304706 0.069398

N -5.448936 1.281513 -0.566691

C -7.457985 2.418513 0.288831

H -7.969667 1.557662 -0.145995

H -7.849865 3.335644 -0.168234

H -7.690412 2.469147 1.359808

C -2.749648 3.865691 0.620782

H -2.187045 4.169527 -0.267585

H -2.036752 3.318894 1.246037

H -3.124548 4.744538 1.152731

S -0.781098 1.627964 -0.859390

C -0.815735 0.053663 -1.770754

H -0.234310 -0.725852 -1.258694

H -0.404190 0.160937 -2.784197

H -1.846286 -0.323513 -1.875285

**[tdzHMeN]^+^**(gasphase)

E = -2.52925933

S -3.686859 1.303745 1.355569

C -3.740086 2.607496 0.180111

N -4.743637 2.503844 -0.686436

C -5.477078 1.421921 -0.452405

N -5.058949 0.677641 0.587128

H -5.488466 -0.180193 0.916178

C -6.666301 1.049610 -1.262647

H -7.128838 0.122180 -0.922795

H -6.366642 0.944785 -2.310499

H -7.400069 1.861040 -1.214177

N -2.861699 3.602390 0.158766

H -2.100700 3.679753 0.815521

H -2.960238 4.316527 -0.550640

**tdzHMeN-SMe** (gasphase)

E = -3.59947657

S -3.443237 1.952609 -0.530239

C -4.073768 0.343647 -1.076205

N -5.343365 0.044930 -1.147998

C -6.275248 0.990132 -0.788271

N -5.900321 2.165781 -0.416538

H -6.665660 2.790490 -0.168552

C -7.705585 0.513933 -0.880621

H -8.411792 1.294324 -0.590256

H -7.849286 -0.359834 -0.236326

H -7.924964 0.192851 -1.904269

N -3.168547 -0.595146 -1.414565

H -2.197471 -0.329145 -1.511785

H -3.518906 -1.437793 -1.841758

S -1.337149 1.696974 -0.646830

C -0.895655 1.099461 1.017396

H 0.197750 1.067947 1.047007

H -1.292101 0.099218 1.203865

H -1.255497 1.794449 1.778637

**MeS^-^** (waterphase)

E = -0.97168186

S 0.000000 0.0000000 -10.347921

C 0.000000 0.0000000 -8.504375

H 0.511102 0.8852540 -8.109939

H -1.022203 0.0000000 -8.109939

H 0.511102 -0.8852540 -8.109939

**[tdzHMe_2_]^+^** (waterphase)

E = -2.76872196

S -7.210716 1.870197 0.000000

C -7.643351 3.526572 0.000000

N -8.951680 3.731802 0.000000

C -9.624114 2.569166 0.000000

N -8.834510 1.493332 0.000000

H -9.151592 0.530102 0.000000

C -11.103922 2.470278 0.000000

H -11.437012 1.432836 0.000000

H -11.498273 2.980457 -0.883866

H -11.498273 2.980457 0.883866

C -6.622708 4.604918 0.000000

H -5.608343 4.205906 0.000000

H -6.763553 5.233974 0.884703

H -6.763553 5.233974 -0.884703

**tdzHMe_2_-SMe** (waterphase)

E = -3.75455273

S -3.444759 1.881847 -0.400465

C -3.987559 0.501699 -1.441112

N -5.226167 0.386534 -1.713391

C -6.283561 1.192138 -1.276354

N -6.615219 2.225277 -1.965808

H -7.459066 2.637814 -1.563826

C -7.028761 0.631715 -0.088160

H -7.834927 1.301610 0.215622

H -6.350444 0.475161 0.756175

H -7.453742 -0.343849 -0.347284

C -2.983949 -0.464741 -1.989963

H -2.476090 -1.001176 -1.183735

H -2.215807 0.058619 -2.565769

H -3.496522 -1.183274 -2.630991

S -1.443003 1.716728 -0.031058

C -1.328037 0.724698 1.490170

H -0.262378 0.728475 1.739057

H -1.659222 -0.300944 1.321547

H -1.893599 1.190602 2.298345

**Cys^-^** (waterphase)

E = -2.80647130

S -1.455212 1.458005 -0.285975

C -1.205830 0.871689 1.437090

C 0.267431 0.764038 1.874093

N 1.082885 1.956956 1.602332

H 0.621111 2.498346 0.869482

H 1.175532 2.540991 2.426256

H 0.282351 0.566818 2.954186

C 0.999695 -0.445261 1.251056

O 0.573734 -1.590543 1.235393

O 2.198688 -0.117033 0.764169

H 2.205617 0.886799 0.962524

H -1.668932 -0.109991 1.570988

H -1.702871 1.560186 2.129507

**tdzHMe_2_-Cys** (waterphase)

E = -5.58088294

S -2.912200 1.399157 -0.863713

C -4.058364 0.161650 -1.533277

N -5.301241 0.431750 -1.555204

C -5.946981 1.587956 -1.101901

N -6.096938 2.580044 -1.905141

H -6.669413 3.293742 -1.450458

C -6.545698 1.452954 0.277919

H -7.032536 2.381245 0.581499

H -5.776340 1.191158 1.010824

H -7.284354 0.644395 0.281124

C -3.536786 -1.139523 -2.057878

H -3.049170 -1.712714 -1.264249

H -2.793803 -0.973336 -2.842589

H -4.369594 -1.718833 -2.458640

S -1.013049 0.652117 -0.914218

C -0.770660 -0.220405 0.692397

C 0.155171 0.500092 1.681587

N -0.240377 1.837792 2.121899

H -0.740575 2.334862 1.390543

H -0.840523 1.797837 2.938267

H 0.196708 -0.142666 2.570872

C 1.627986 0.559174 1.207569

O 2.206835 -0.343875 0.629285

O 2.229478 1.700373 1.548629

H 1.476083 2.212332 1.980518

H -0.354149 -1.198693 0.443033

H -1.752684 -0.368069 1.142540

**tdzMe_2_** (waterphase)

E = -2.79380495

S -3.306264 0.856981 0.000000

C -3.674820 2.536384 0.000000

N -4.978754 2.730934 0.000000

C -5.628890 1.524403 0.000000

N -4.904562 0.414024 0.000000

C -7.124946 1.473830 0.000000

H -7.476272 0.441501 0.000000

H -7.521818 1.986337 -0.882636

H -7.521818 1.986337 0.882636

C -2.650024 3.620201 0.000000

H -1.636493 3.216050 0.000000

H -2.776286 4.253529 0.884310

H -2.776286 4.253529 -0.884310

**[tdzMe_2_-SMe]^-^** (waterphase)

E = -3.69570014

S -3.797804 0.159167 0.755098

C -4.393217 -0.650103 -0.797132

N -5.484036 -0.260154 -1.305884

C -6.306473 0.885627 -0.777313

N -6.080096 2.065265 -1.108751

C -7.473580 0.311111 0.034000

H -8.102249 1.126311 0.400895

H -7.124272 -0.283511 0.887045

H -8.085846 -0.353111 -0.590264

C -3.522512 -1.700043 -1.426371

H -3.382267 -2.557639 -0.762515

H -2.527953 -1.301592 -1.654200

H -3.994094 -2.040623 -2.350244

S -2.200487 -0.870217 1.515793

C -2.929284 -2.152741 2.583375

H -2.074591 -2.640949 3.061928

H -3.485088 -2.886475 1.996849

H -3.567714 -1.703692 3.346169

**[tdzHMeN]^+^** (waterphase)

E = -2.62544960

S -5.254224 2.727143 1.031485

C -3.733861 2.630390 0.181184

N -3.547066 1.470469 -0.457689

C -4.592874 0.662841 -0.279575

N -5.571354 1.168359 0.473774

H -6.436407 0.700693 0.715126

C -4.684672 -0.698198 -0.869704

H -5.598311 -1.207323 -0.563813

H -3.817342 -1.286499 -0.557501

H -4.661470 -0.619885 -1.960840

N -2.853568 3.620258 0.174622

H -1.989638 3.508533 -0.334743

H -3.014818 4.484096 0.666508

**tdzHMeN-SMe**(water phase)

E = -3.61180454

S -3.302266 2.016547 -0.663162

C -4.008356 0.448840 -1.261836

N -5.287604 0.180284 -1.264873

C -6.255515 1.091043 -0.896116

N -6.023259 2.361569 -0.826126

H -6.864359 2.860488 -0.531792

C -7.599113 0.450931 -0.628144

H -8.352378 1.195155 -0.363936

H -7.512718 -0.276164 0.186738

H -7.934825 -0.099273 -1.513522

N -3.139799 -0.464912 -1.707484

H -2.146340 -0.287286 -1.676444

H -3.479355 -1.368591 -1.999577

S -1.262599 1.677572 -0.517886

C -1.030124 0.983870 1.149423

H 0.053241 0.886617 1.266021

H -1.495748 0.000966 1.235969

H -1.419684 1.667171 1.905438

**tdzMeN**(waterphase)

E = -2.64447836

S -3.717439 1.264174 1.259194

C -3.709401 2.595907 0.134037

N -4.702469 2.495946 -0.735399

C -5.423571 1.363661 -0.469145

N -5.067629 0.582823 0.536295

C -6.609110 1.022789 -1.319639

H -7.070939 0.093625 -0.984080

H -6.306594 0.913799 -2.366517

H -7.351601 1.826686 -1.275646

N -2.780230 3.574059 0.118317

H -2.188109 3.707962 0.922675

H -2.978655 4.397748 -0.431145

**[tdzMeN-SMe]^-^** (waterphase)

E = -3.54828303

S -3.513213 2.166042 -0.870607

C -3.997740 0.481008 -1.458203

N -5.209514 0.110305 -1.391208

C -6.318378 1.004630 -0.909958

N -6.887288 1.843918 -1.636099

C -6.680302 0.580308 0.521975

H -7.509534 1.188512 0.892040

H -5.827761 0.685275 1.204943

H -6.978659 -0.476242 0.547823

N -2.966007 -0.311868 -1.903941

H -2.140243 0.169588 -2.236992

H -3.256510 -1.087175 -2.485887

S -1.480786 2.361229 -0.996906

C -0.832993 1.699150 0.570908

H 0.245479 1.880181 0.529080

H -1.021138 0.627441 0.650774

H -1.261458 2.234665 1.419643
